# Acoustofluidics: Technology Advances and Applications from 2022 to 2024

**DOI:** 10.1021/acs.analchem.4c06803

**Published:** 2025-03-25

**Authors:** Toktam Godary, Brandi Binkley, Zhengru Liu, Olanrewaju Awoyemi, Amanda Overby, Herbi Yuliantoro, Bethany J. Fike, Sydney Anderson, Peng Li

**Affiliations:** C. Eugene Bennett Department of Chemistry, West Virginia University, Morgantown 26506-6201, West Virginia, United States

Acoustofluidics, the interplay of acoustics and fluid dynamics, has experienced exponential growth in the past decade. Acoustic waves are the result of the vibration or oscillation of the particles in the medium through which they propagate. These waves not only propagate through the medium but also exert forces on it and on objects within their pathway. Through careful engineering of wave generation and propagation, often in conjunction with microfluidic platforms, acoustofluidics enables the precise manipulation and control of a wide range of objects at the microscale, including biological objects and fluids. Two typical types of acoustofluidic devices are bulk acoustic wave (BAW) devices and surface acoustic wave (SAW) devices, classified based on the mode of wave propagation. A typical BAW device includes a piezoelectric transducer and a resonant chamber, while SAW requires patterned electrodes on top of a piezoelectric substrate. Both types have demonstrated extraordinary versatility and effectiveness, serving as powerful tools across diverse application areas such as biomedical diagnostics, materials synthesis, and tissue engineering. This review highlights the major advancements in acoustofluidics spanning from January 2022 to November 2024, emphasizing both technological innovations and the expansion of application domains. By showcasing the breadth of the field and the ingenuity behind its numerous breakthroughs, we aim to provide readers with a comprehensive understanding of the current state of acoustofluidics and its future directions.

## Advances in Technology Development

### Device Fabrication

Fabricating methods are essential to the functionality and commercialization of acoustofluidic devices. While standard microfabrication procedures still apply, factors such as the acoustic field in the channel, coupling layer, matching the acoustic wavelength with device geometry, and material properties are unique to acoustofluidic devices.

Channel materials have a major impact on the acoustic field and energy efficiency of acoustofluidic devices. Acikgoz et al.^1^ evaluated the effectiveness of various materials for use as chip materials in acoustofluidic particle and cell manipulation. The materials studied include silicon, glass, epoxy with fiberglass filling (FR4), polydimethylsiloxane (PDMS), and poly(methyl methacrylate) (PMMA). The study shows that while PDMS and PMMA are the most suitable for biological purposes, silicon is best for high precision and structural stability, and glass is good when optical clarity is needed. Luzuriaga et al.^2^ reported that acoustophoretic motion of polystyrene particles can be observed in a hybrid millifluidic resonator. The resonators have channels that are embedded in hydrogel with properties similar to liquids. Fakhfouri et al.^3^ fabricated a SAW device made of dry film resist (DFR), which improves the precision and reproducibility of microchannels. By generating acoustic streaming that complements the acoustic radiation effect, their device was able to trap nanoparticles as small as 200 nm.

To generate a bulk acoustic wave (BAW), Fuchsluger et al.^4^ reported that lateral vibration modes of a plate transducer can also be used in an acoustic resonator device. These lateral plate transducer modes were demonstrated to perform rapid particle focusing when excited at a frequency of 540 kHz. Qiu^5^ studied the potential of using lead-free piezoelectric materials to BAW devices. Using the common transducers that are made of lead zirconate titanate results in environmental and biocompatibility issues. Through experiments and simulations, the researcher demonstrated that lead-free transducer devices can match the typical lead transducer devices at a low power and are even better at intermediate powers. Qiu et al.^6^ studied the impact of transducer position on the energy efficiency of BAW resonator. They found that placing the transducer on the side for actuation led to a 4-fold increase in energy density, due to acoustic field symmetry breaking and the improvement in energy conversion efficiency.

**Figure 1:**
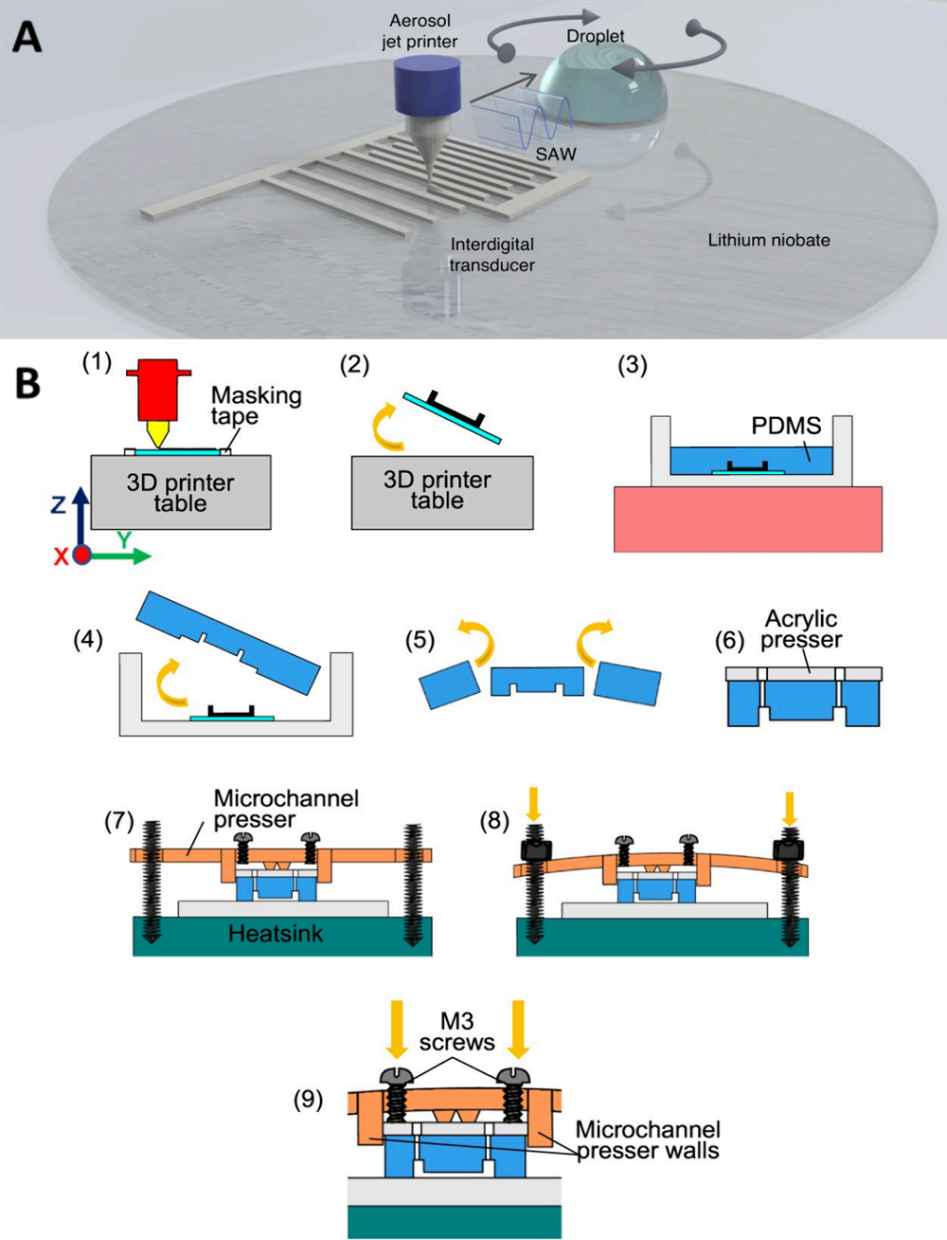
(A) Aerosol jet printing to fabricate IDTs. Reprinted by permission from Macmillan Publishers Ltd.: NATURE, Rich, J.; Cole, B.; Li, T.; Lu, B.; Fu, H.; Smith, B. N.; Xia, J.; Yang, S.; Zhong, R.; Doherty, J. L.; Kaneko, K.; Suzuki, H.; Tian, Z.; Franklin, A. D.; Huang, T. J. Microsystems & Nanoengineering 2024, 10 (1), 2. (ref (7)). Copyright 2024 under a Creative Commons Attribution 4.0 International License (http://creativecommons.org/licenses/by/4.0/). (B) Reusable IDT and microchannel fabrication by using a thin layer of polyamide. Reprinted by permission from IOP Publishing, Mikhaylov, R.; Martin, M. S.; Dumcius, P.; Wang, H.; Wu, F.; Zhang, X.; Akhimien, V.; Sun, C.; Clayton, A.; Fu, Y.; Ye, L.; Dong, Z.; Wu, Z.; Yang, X. A Reconfigurable and Portable Acoustofluidic System Based on Flexible Printed Circuit Board for the Manipulation of Microspheres. *J. Micromechanics Microengineering***2021**, 31 (7), 074003. 10.1088/1361-6439/ac0515. (ref (16)). Copyright 2021 under a Creative Commons Attribution 4.0 International License (http://creativecommons.org/licenses/by/4.0/).

Using interdigital electrodes (IDTs) is still the most dominant way of generating surface acoustic waves (SAWs) in acoustofluidic devices. While IDTs can be fabricated through the standard “lift-off” procedure, it is a lengthy process and requires access to clean room facilities. Rich et al.^7^ reported fabricating IDTs using aerosol jet printing (Figure 1A). They successfully printed SAW IDTs using different materials including silver nanowires, graphene, and poly(3,4-ethylenedioxythiophene) polystyrenesulfonate (PEDOT:PSS). The devices were then demonstrated to be comparable to clean room fabricated devices for acoustic streaming generation and particle concentration. Wang and Qian^8^ used femtosecond laser micromachining to fabricate SAW devices. This method generates the IDTs by micromachining steel foil to form a mask and then directly evaporating metal on a piezoelectric substrate using the mask. The resulting device was demonstrated for various acoustofluidic functionalities. Zhang et al.^9^ introduced a novel IDT design termed unapodization. Unapodization removes the apodization effect, creating laterally uniform surface acoustic waves and resulting in reduced variations and smoothed-out wave amplitudes across the surface.

Surface properties have a major impact on acoustofluidic particle and droplet manipulation. Ning et al.^10^ reported using self-assembled monolayers (SAMs) of 1*H*,1*H*,2*H*,2*H*-perfluorododecyltrichlorosilane (FDTS) on LiNbO_3_ substrates to enhance SAW microfluidic performance. FDTS SAMs were deposited onto the LiNbO_3_ substrates via chemical vapor deposition, creating a hydrophobic surface. Then these fabricated devices were compared to untreated SAW devices, and it was found that there was an increase in the droplet driving speed which therefore enables faster and more precise liquid jetting. Al-Ali et al.^11^ reported using 3-(trimethoxysilyl) propyl methacrylate (TMSPMA) silane treatment on PDMS to enhance its attachment to LiNbO_3_ substrates.^7^ The surface modification strategy involved using oxygen plasma treatment to alter the surface chemistry of the PDMS improving its ability to bond with other materials.

Due to the cost and lengthy fabrication procedures of fabricating SAW substrates, there is a growing need for developing reusable SAW devices. Kuruoglu^12^ reported a strategy to detach the permanent bonding between the LiNbO_3_ substrate and PDMS. They found immersing the device in 0.1 M potassium hydroxide solution for 2 h can effectively detach the device and no significant deterioration in the microfluidic channel or LiNbO_3_ surface after 3 cycles of bonding-detachment. Another common strategy to achieve reusable SAW is separating the SAW substrate and the fluidic channel, which necessitates an efficient coupling layer. Park et al.^13^ studied the impact of the thickness of the PDMS coupling layer on the various acoustofluidic phenomena. They found that when the thickness is >8 times the wavelength of the acoustic wave, the coupling layer will absorb almost all the energy and generate heat. Therefore, to ensure efficient acoustic streaming and acoustic radiation force generation in the microchannel, the thickness of the PDMS layer needs to be controlled. Kolesnik et al.^14^ also investigated the optimal coupling layer and superstrate thickness. Results indicated that a superstrate thickness 0.55 times the acoustic wavelength results in maximized acoustic coupling. Chang et al.^15^ explored using *n*-dodecane as a coupling layer to enhance the performance of reusable acoustofluidic microchips. With viscosity similar to water but a lower surface tension and higher boiling point, *n*-dodecane offers unique properties. These properties allow the *n*-dodecane to reduce the friction between the microchip and fluid. *n*-Dodecane also enhances the transfer of acoustic energy from the surface to the fluid, improving particle manipulation. Yang and co-workers^16^ reported fabricating reusable IDTs by mechanically clamping interdigital electrodes (IDEs) patterned on a printed circuit board (PCB) onto a piezoelectric substrate. They achieved reusability for both IDTs and the PDMS microchannel. A thin layer of polyamide was used to directly fabricate the microchannel onto the printed circuit board (Figure 1B). This allows for the formation of flexible structures that can be adapted for different applications.

Fabricating IDTs on a flexible piezoelectric substrate is the first step to achieve acoustofluidics for wearable devices and many *in vivo* applications. Zahertar et al.^17^ reported flexible acoustic wave devices that were fabricated on substrates that consist of thin layers of metal and polymer. Overall, the results indicated that the combination of metallic and polymer layers offers unique advantages. Metallic layers are ideal for acoustic wave generation while the polymer layers provide flexibility and allow for tuning of the device properties. Pang et al.^18^ demonstrated that both Rayleigh waves and shear horizontal SAWs can be generated simultaneously in an inclined ZnO film. The two wave types can work together to enhance particle manipulation and fluid control in devices with the dual wave approach also providing a more versatile platform.

## Advances in Particle Manipulation

Acoustics has been demonstrated as an excellent tool for manipulating both biological and nonbiological particles in various fluids via a contactless manner. Acoustofluidic particle manipulation is mainly achieved by controlling the acoustic radiation force (ARF) exerted on the particle. Therefore, accurate characterization of ARF under various experimental conditions is important. Liu et al.^19^ proposed a lookup table for determining the acoustic radiation force by examining the particle acoustophoresis mode in discrete phase modulated standing SAW fields. Edthofer et al.^20^ employed two-step focusing to measure the relative acoustic mobility of polystyrene beads by experiments. The variations in acousto-mechanical properties of the beads could be quantitatively analyzed, which showed a large spread in their material properties, indicating that extra normalization may be necessary when using different polystyrene particles for device calibration.

### Focusing

Focusing particles under a continuous flow is one of the early demonstrations for acoustofluidic particle manipulation. Recent studies have focused on expanding the flexibility for different targets. Park et al.^21^ developed an acoustic focusing device with a square microchannel and a single acoustic actuation source to align and focus nonspherical cells, such as ellipsoidal *Euglena gracilis*. The device achieved 96% orientation efficiency and a focusing width under 7.8 μm at flow rates up to 200 μL/min. Hammarstrom et al.^22^ reported a protein microcrystal focusing device for real-time monitoring during serial crystallography, achieving up to a 200-fold concentration increase. Wang et al.^23^ developed a microflow cytometer that allows for particle focusing via acoustic streaming tunnel and fluorescence enhancement via a microreflector for detection to occur simultaneously without sheath fluid. The microreflector improved the fluorescence intensity of AlN-Mo substrate by 1.8 and 8.3-fold over that of the Si and SiO_2_ substrates, respectively. They further found that different size particles had different velocities when they exited the transducer region. Based on this phenomenon, this system was also able to differentiate particle size based on their transit time through the detection region.

Another important area for acoustic focusing is to improve the focusing performance for submicron particles. Hemptinne et al.^24^ utilized frequency-sweeping techniques to achieve efficient particle focusing across multiple parallel channels, overcoming the influence of structural variabilities on the resonance for each parallel channel. In acoustic focusing, acoustic streaming-induced drag force could act against acoustic radiation force for focusing. When the particle size is large, ARF dominates over the drag force. However, when the particle size is small (<1 μm), the drag force could become so significant that it disrupts particle focusing. Gerlt et al.^25^ reported focusing 1 μm polystyrene particles in round glass capillaries (500 μm ID) at a flow rate of 5 μL/min. Their simulation results indicated that operating frequencies that are near the optimal resonance frequency can effectively reduce the acoustic streaming while maintaining sufficient ARF for particle focusing. Similarly, Lee and co-workers^26^ induced a unidirectional vortex streaming pattern in a 100 μm square capillary and successfully focused 0.25 μm polystyrene particles at a flow rate of 2 μL/min. In the following work, they used a smaller square capillary (50 μm) and higher actuation frequency (14.9 MHz) and achieved focusing of 100 nm polystyrene beads at a flow rate of ∼2 μL/min.^27^ Harshbarger et al.^28^ implemented an optical feedback system to achieve automatic optimization of focusing. When compared with experienced scientist operators, the automatic feedback control system achieved superior performance, achieving focusing of 600 nm particles using a BAW resonator by selecting the optimal working frequency. Devendran et al.^29^ applied diffractive-acoustic SAW (DASAW) for improving the control of small particles. The author found that DASAW configuration could reduce critical particle size by reducing the inherent streaming effects while a similar acoustic radiation field could be maintained.

### Sorting

Richter et al.^30^ reported an acoustic activated absorbance droplet sorter for the sorting of tartrazine dye droplets at different concentrations (30 and 80 μM). Biconcave microlenses were integrated into the microfluidic device to improve the detection, and traveling SAW (TSAW) was used for droplet deflection achieving a sorting rate of 1000 droplets/s. Nawaz et al.^31^ utilizes focused traveling surface acoustic waves (FTSAW) and real-time deformability cytometry (RT-DC) to sort cells by their physical properties in high throughput. The focused interdigital transducer (FIDT)-based sorting RT-DC platform (soRT-DC) was tested with different white blood cells from TBC-depleted blood and raw diluted whole blood, successfully sorting the cells based on size with a purity greater than 92% and a cell viability greater than 90%. Similarly, Sethia et al.^32^ utilized TSAWs and image-based detection to sort human stem cell-derived β cell clusters (SC-β cell clusters) by size. SC-β cell clusters ranging from diameters of ∼100–500 μm were passed through the inlet of the device, and pictures of the clusters were taken with a 2× magnification microscope. For a separation cutoff of 250 μm, this setup gave a sorting efficiency of 78–90% with a throughput of up to 0.2 SC-β cell clusters/s. Fuchsluger et al.^33^ introduced a microfluidic sorter with a geometrically defined acoustic active region using BAW to selectively focus particles of interest into a central stream while leaving unwanted particles unaffected.

### Separation

Acoustofluidics is also a powerful tool for both label-free and label-based particle separation. To date, it has been demonstrated to separate various kinds of particles including mammalian cells, bacteria, viruses, and extracellular vesicles. For label-free separations, it is possible to separate cells acoustically based on their differences in size, density, and compressibility. Wu et al.^34^ explored power-controlled SAW systems for microparticle separation, utilizing standing surface acoustic waves (SSAWs) and power monitoring to optimize separation efficiency. Their approach demonstrated good agreement between experimental results and theoretical predictions for separating microparticles of varying sizes. During the past 3 years, many reports focused on the optimization of the tilted-angle standing surface acoustic wave (taSSAW) device for cell separation. taSSAW takes advantage of the angle between the flow direction and the SSAW field to overcome the separation distance limit in conventional acoustofluidic separation devices. Several groups reported numerical analysis of key design parameters of taSSAW including tilt angle, prefocusing width, and aperture length of IDTS for improved separation performance including submicron particles.^35,36^ Wu et al.^37^ combined bipolar electrode-based DEP focusing with taSSAW to separate cells based on size and mechanical properties. This integrated device achieved separation efficiency of 94% and purity of 92% for polystyrene particles and maintained viability for THP-1 and yeast cells. Xue et al.^38^ employed a divergent microchannel in the taSSAAW device to improve the separation resolution. They achieved a 91% separation success rate of myelogenous leukemia cell line K562 and the natural killer cell line NK92, which have similar size and density but different compressibility. Peng et al.^39^ numerically studied a taSSAW design that employs a spiral channel for cell/particle focusing with taSSAWs, indicating the potential of further increasing separation distance for particles/cells with similar properties.

**Figure 2:**
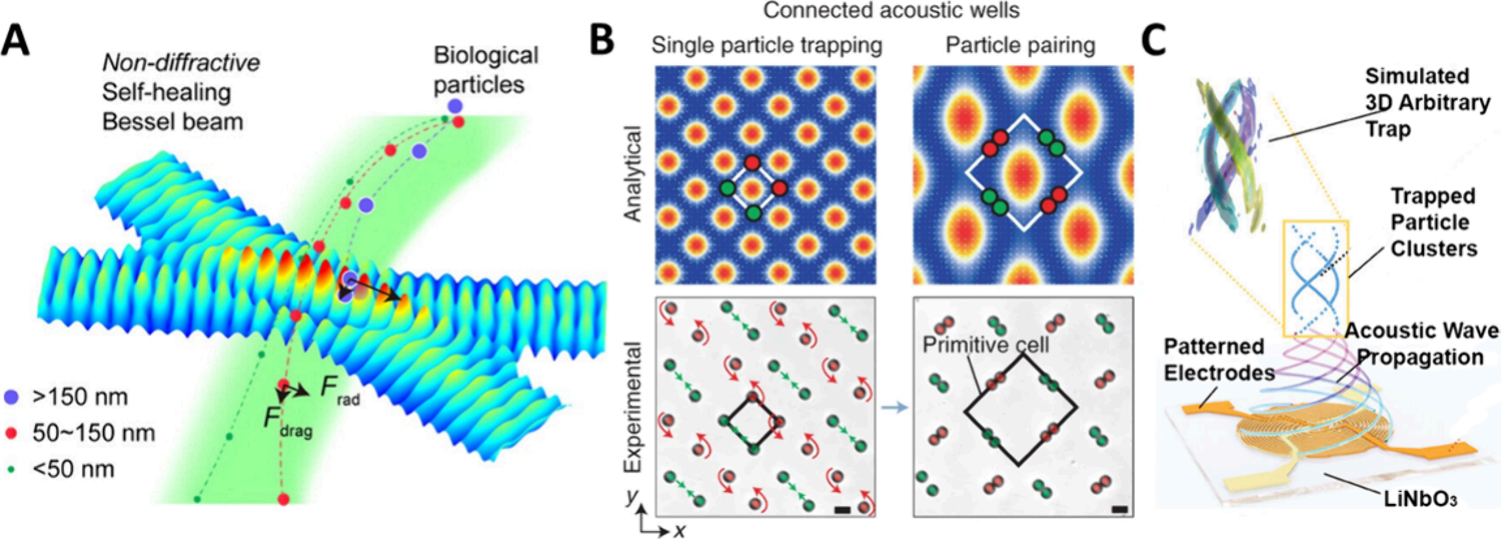
(A) Using IDTs to create a Bessel beam region in the device resulting in improved nanoparticle separation. Reproduced from Xia, J.; Wang, Z.; Becker, R.; Li, F.; Wei, F.; Yang, S.; Rich, J.; Li, K.; Rufo, J.; Qian, J.; Yang, K.; Chen, C.; Gu, Y.; Zhong, R.; Lee, P. J.; Wong, D. T. W.; Lee, L. P.; Huang, T. J. Acoustofluidic Virus Isolation via Bessel Beam Excitation Separation Technology. *ACS Nano* 2024, 18 (33), 22596–22607. (ref (40)). Copyright 2024 American Chemical Society. (B) Cell pairing and separation using Fourier synthesized harmonic waves which allow for high resolution tuning of the acoustic field. Reprinted by permission from Macmillan Publishers Ltd.: NATURE, Yang, S.; Tian, Z.; Wang, Z.; Rufo, J.; Li, P.; Mai, J.; Xia, J.; Bachman, H.; Huang, P.-H.; Wu, M.; Chen, C.; Lee, L. P.; Huang, T. J. Harmonic Acoustics for Dynamic and Selective Particle Manipulation. Nat. Mater. 2022, 21 (5), 540–546. (ref (53)). Copyright 2022. (C) BEACON platform that uses IDT patterns for the generation of acoustic holograms to be used for patterning. Reproduced from Acoustography by Beam Engineering and Acoustic Control Node: BEACON. Yu, W.; Zhu, H.; Upreti, N.; Lu, B.; Xu, X.; Lee, L. P.; Huang, T. J. *Advanced Science* 2024, 2403742. (ref (59)) Copyright 2024 under a Creative Commons Attribution 4.0 International License (http://creativecommons.org/licenses/by/4.0/).

In addition to taSSAW, several new designs were reported to improve the separation performance for submicron particles. Xia et al.^40^ designed Bessel interdigital transducers (BIDTs) to create a Bessel beam region in the fluidic channel (Figure 2A). Compared with conventional SAW devices, this design achieved ∼5 times higher acoustic intensities in the Bessel region, leading to improved performance for nanoparticle separation. They demonstrated the separation of 30, 100, and 400 nm particles in one device, and the successful enrichment of SARS-CoV-2 virus particles from saliva samples. Zhang et al.^41^ leveraged the leaky wave generated by a pair of standard IDTs to form acoustic virtual pillars in a microfluidic channel. By employing the optimal channel thickness and excitation frequency, a traveling leaky wave that is orthogonal to the original SAW direction can be formed, leading to the formation of acoustic virtual pillars along the channel direction. Bigger particles experience bigger deflection forces as they pass the virtual pillar. After this repeated cycle, separation of nanoparticles can be achieved. They demonstrated the enrichment of small exosomes (60–80 μm) from larger ones (90–150 μm) with a purity of 96%. Yang et al.^42^ employed acoustic streaming induced by a gigahertz BAW resonator to achieve nanoparticle separation. They designed an arc-shape resonator that can induce strong acoustic streaming at the edge of the resonator. Combining the lateral fluid flow and the trapping effect of microvortices, the device was able to capture 30 nm polystyrene particles, while directing 150 nm particles to the desired outlet. Ang et al.^43^ introduced a glass ceiling to the PDMS-based SAW nanosieve device. Due to the high acoustic impedance of glass, it reduced the loss of acoustic energy through the PDMS ceiling thereby improving the energy efficiency of the device. Their results showed a 30-fold improvement in throughput over the PDMS device without the glass ceiling.

For the size-based separation, Khan et al.^44^ employed TSAWs to generate differential acoustic radiation forces and torques, enabling the separation of spherical and prolate particles with high recovery and purity. Undvall et al.^45^ reported the effect of inertial forces on acoustofluidic particles when separation is performed at high flow rates. They showed that the spillover effect, where particles diverge from their proposed path and spill out through the center outlet, could lead to the failure of acoustic particle separation.

In addition to exploiting the differential mobility induced by ARF, isoacoustic focusing (IAF) has also been reported to achieve cell separation in an acoustic field. This separation utilizes the inhomogeneous fluid and separates cells not based on their size but their acoustic impedance. Rezayati Charan^46^ studied IAF in the stop-flow regime to achieve precise characterization of neutrophils and K562 cancer cells using acoustic impedance gradients and proposed an effective gradient for separating these cells with very similar properties.

### Patterning

Over the years, acoustofluidic methods have gained tremendous success in arranging randomly distributed cells or particles into lines or arrays in a biocompatible and contactless manner. Traditionally, it is achieved via creating various patterns of standing acoustic wave fields. One classical setup involves positioning 2 pairs of IDTs orthogonally to each other to generate various grid-like patterns. Konig and co-workers^47^ studied the 3D particles patterns generated by a 2D SSAW field, indicating further investigation for the pattern results is needed as particles with positive acoustic contrast were found to be present at both pressure nodes and antinodes. They also studied the thermal effects induced by the 2D SSAW device, where careful control of operational parameters is needed to ensure maximum biocompatibility for SSAW-based cell manipulation.^48^ In addition to SSAW-based patterning, Huang et al.^49^ reported dynamic control of particle patterns in an octagonal chamber. This device employed eight piezoelectric transducers to generate ultrasonic fields that produce complex acoustic patterns such as stripes, lattices, and hexagons by adjusting the phase parameters. Further advances in particle manipulation methods focus on harnessing novel geometries to improve pattern complexity and control. Tang et al.^50^ proposed a fluidic chamber with Sierpiński-carpet-inspired fractal designs, which, when combined with uniform acoustic fields, resulted in sophisticated patterning that could be scaled for diverse applications such as micromachine orientation and biological sample handling. Placing a capillary on a SAW substrate is a convenient way to rapidly pattern particles and cells. Maramizonouz et al.^51^ studied the impact of the direction and different cross sections of capillary tubes on the acoustic field inside a capillary. Pei et al.^52^ combined FIDTs with a capillary to achieve particle enrichment, alignment, and transport.

While conventional acoustofluidic patterning methods are efficient in pushing particles together, they cannot separate the particles afterward. Yang et al.^53^ overcame this limit by employing Fourier-synthesized harmonic waves to achieve high-resolution tuning of an acoustic field inside a chamber. They achieved high-throughput cell pairing and separation by applying sequential nanosecond pulse of SAW (Figure 2B). Finally, this method was demonstrated to study the intercellular adhesion strength in a high-throughput manner.

Another focus for acoustofluidic patterning is achieving complex and arbitrary patterns to meet the needs of various applications. One strategy leverages a microfabricated structure to overcome the limitations of a simple standing acoustic wave field. Harley et al.^54^ introduced subwavelength microresonators with spring-mass designs that amplify acoustic oscillations for submicron-scale manipulation. These resonators, fabricated using 3D microprinting and integrated into microfluidic chambers, produce localized evanescent fields, enabling arbitrary particle patterns based on the design of microstructures. Kolesnik et al.^55^ introduced an “acoustic stencil” created by the combination of a half-wavelength acoustic field and structure surface with different cavities. This method enabled micropatterning of polystyrene microparticles and mammalian cells with complex patterns. Li et al.^56^ reported a detachable acoustofluidic device for creating complex particle patterns. They utilized a microfabricated silicon waveguide to control the coupling acoustic waves with the PDMS chamber. Due to its detachable setup, this method allows for the possibility of achieving different patterns with the same substrate by swapping the waveguides with different patterns. Pan et al.^57^ leveraged reconfigurable magnetic micropillar arrays to achieve dynamic manipulation of particles. Under the influence of an external magnetic field, magnetic particles can form micropillars in a fluid chamber, which was subsequently activated by SAW to trap particles around the pillar. Zhang et al.^58^ achieved complex particle patterning utilizing photoacoustic effects. They employed a laser to generate localized Lamb wave fields for particle manipulation on the membrane surface. Since the laser pattern can be arbitrarily controlled, the patterns can also be dynamically controlled to allow creating animation of particle patterns.

In addition to using microfabricated structures, acoustic holography has gained significant traction for achieving tunable and arbitrary particle manipulation during the past several years. Yu et al.^59^ presented the Beam Engineering and Acoustic Control Node (BEACON) platform, utilizing specific and high-resolution (<1 μm) IDT patterns to generate configurable 2D and 3D acoustic holograms. This system achieved high spatial resolution (∼25 μm) and complex 3D particle patterns (Figure 2C). Xu et al.^60^ reported programmable acoustic holography that is modulated by the sound speed of the coupling layer fluid. They utilized a fluid layer to couple the 3D printed hologram phase plate with a chamber filled with PDMS particles. The acoustic holography visualizes the pattern of PDMS particles. By changing the liquid to one with a different sound speed in the fluid layer, different patterns of PDMS particles can be achieved. In follow-up work, Xu et al.^61^ also reported that the 3D printed hologram phase plate can be combined with a detachable microfluidic channel to achieve complex patterns in microchannels. Melde et al.^62^ employed multiple transducers and 3D printed holograms to achieve one-step assembly of 3D patterns of microparticles, hydrogel beads, and biological cells. The resulting pattern can be fixed via the gelation of the surrounding medium.

Phased array transducers have also been used to generate complex patterns in 3D space. Hirayama et al.^63^ created a volumetric display based the acoustic holography particle trapping. In recent work, they implemented a fast algorithm to account for the acoustic wave scattering caused by physical objects present in the patterning space. This method enabled a volumetric display that could interact with physical objects above or below it. To improve the operation of phased array transducers, Fushimi et al.^64^ reported a digital twin approach to accelerate the optimization of acoustic hologram. This approach involves integrating experimental measurement with numerically obtained derivatives of the loss function to improve the loss function.

### Particle Trapping and Actuation

Gao et al.^65^ developed an acoustofluidic device that employs acoustic bubble for trapping, rotating, and culturing tumor spheroids. This device achieved 91% trapping efficiency and could form viable spheroids within 30 min. After spheroids formation, a long-term culture was also demonstrated with 84% cell viability after 3 days. Richard et al.^66^ presented an acoustofluidic trapping device that utilized focused IDTs for cell trapping and release, integrating an external valve to guide the cells’ trajectory. The device allowed for the controlled dispensing of single cells or small clusters, maintaining cell viability and adhesion.

### Acoustic Tweezers

**Figure 3:**
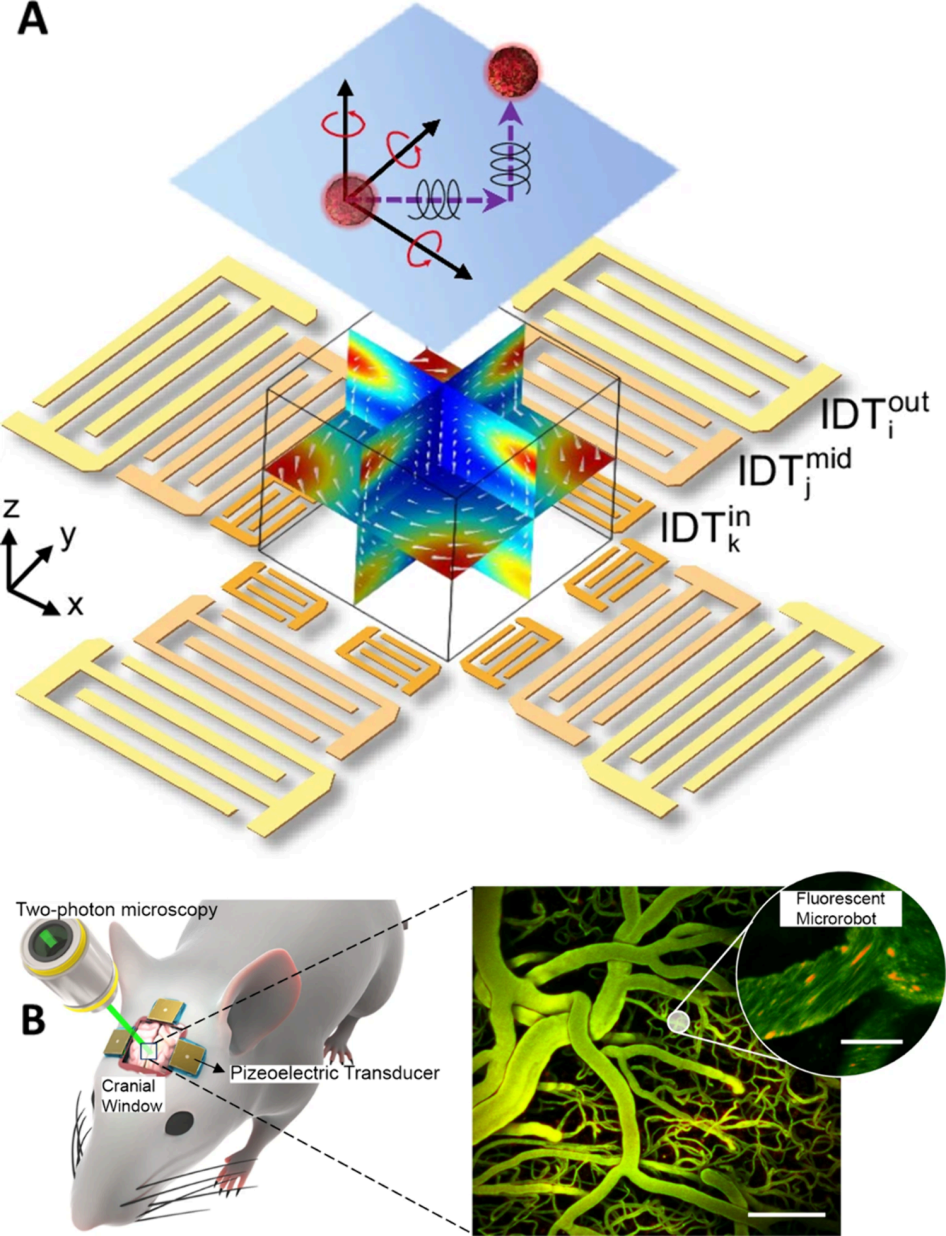
(A) Six degrees of freedom particle manipulation by joint subarray acoustic tweezers (JSAT). Reprinted by permission from Macmillan Publishers Ltd.: NATURE, Shen, L.; Tian, Z.; Yang, K.; Rich, J.; Xia, J.; Upreti, N.; Zhang, J.; Chen, C.; Hao, N.; Pei, Z.; Huang, T. J. Joint Subarray Acoustic Tweezers Enable Controllable Cell Translation, Rotation, and Deformation. *Nat. Commun*. 2024, 15 (1), 9059. (ref (67)) Copyright 2024 under a Creative Commons Attribution 4.0 International License (http://creativecommons.org/licenses/by/4.0/). (B) Using acoustically activated robots to navigate through vasculature networks in a mouse brain. Reprinted by permission from Macmillan Publishers Ltd.: NATURE, Del Campo Fonseca, A.; Glück, C.; Droux, J.; Ferry, Y.; Frei, C.; Wegener, S.; Weber, B.; El Amki, M.; Ahmed, D. (Enlarged detail) Ultrasound Trapping and Navigation of Microrobots in the Mouse Brain Vasculature. *Nat. Commun*. 2023, 14 (1), 5889. (ref (81)). Copyright 2023 under a Creative Commons Attribution 4.0 International License (http://creativecommons.org/licenses/by/4.0/).

Another important particle manipulation capability for acoustofluidics is manipulating single or a small amount particles, which is often termed acoustic tweezers. Shen et al.^67^ developed Joint Subarray Acoustic Tweezers (JSAT), which provide six degrees of freedom manipulation, enabling simultaneous translation and rotation of cells. They designed 3 layers of IDTs on the LiNbO_3_ substrate to achieve complex control of cells and particles (Figure 3A). The outer layer IDTs were used to generate patterns of SSAW field for controlling the cell movement in the chamber based on ARF. The middle layer IDTs were used to generate SSAW acoustic streaming vortices to control the rotation of particles along x and y direction. Finally, the inner layer of IDTs was used to generate TSAW acoustic streaming for controlling the rotation along *z* axis. Wang et al.^68^ employed 3 pairs of IDTs that were symmetrically positioned around a center fluid chamber. By independently adjusting the TSAW generated from these IDTs, clockwise and anticlockwise rotation, as well as linear motion of particles, were demonstrated. Shen et al.^69^ combined SAWs with dielectrophoresis (DEP) to improve the flexibility of particle manipulation. This setup overcomes the SAW-based tweezers’ inability to direct different particles to different paths at the same time thereby demonstrating cyclical cell pairing and separation. In addition, the combination of SAW and DEP opens the possibility of distinguishing cell types based on their mechanical and electrical properties. Xu et al.^70^ developed an acoustic ring resonator (RR) for manipulating microparticles along the waveguides and the RR. The acoustic RR exhibited a high Q factor (>3000), achieving energy-efficient particle manipulation. By changing the design of the waveguides, different particle trajectories can be achieved.

In addition to SAW-based acoustic tweezers, BAW has also been demonstrated for acoustic tweezers, especially for *in vivo* manipulation. You et al.^71^ developed a GHz BAW resonator array that exploits acoustic streaming forces for controlling the movement of particles. Due to the short attenuation distance of GHz acoustic waves, strong acoustic streaming can be induced with minimum interference by ARF. By controlling the input power amplitude, particles can move along complex paths in a fluid chamber. Li et al.^72^ combined piezoelectric ring transducers with 3D printed acoustic holography plates to generate vortex acoustic beam to trap particles in 3D space. The chirality of the vortex beam can be adjusted to control the rotation of particles. Coupled with a robotic arm, the trapped particle can be transported in an arbitrary path in 3D space. They also demonstrated that this method works even through biological barriers, e.g., a tissue layer. Jooss et al.^73^ demonstrated controlling the motion of microbubbles inside a zebrafish that was fixed by agar gel. The axial movement of microbubbles along the zebrafish was achieved by adjusting the frequency of four piezoelectric transducers that were placed orthogonally. In 2023, Shapiro’s group and Zheng’s group both reported the strategy of using gas vesicles (GVs) to achieve *in vivo* acoustic manipulation.^74^ Cells transfected with genes for expressing GVs exhibit significantly higher acoustic contrast compared with background, which enables in vivo acoustic tweezers manipulation using ultrasound.^75^ Yiannacou et al.^76^ introduced a programmable microfluidic platform that employs machine vision to predict and adjust acoustic fields in real-time. The adaptive controller utilizes online learning and machine vision to optimize acoustic fields for particle trajectory control, significantly increasing manipulation accuracy. The inclusion of automation reinforces the scalability and robustness of acoustic tweezers, linking their applications across fields like biophysics and material science.

### Microrobots

Acoustically driven microrobot/microswimmer is an emerging area that has gained significant interest in recent years. These devices can be activated through the interaction with acoustic waves, which generate sufficient propulsion for traveling in various mediums. One widely used strategy is fabricating microstructures with sharp edges/corners to achieve propulsion induced by acoustic streaming. Liu et al.^77^ developed an acoustic driven microswimmer featuring an elliptical main body with asymmetric, flexible tails that oscillate under acoustic excitation. By tuning the resonant frequencies of the tails, the swimmer achieves real-time directional control, transitioning seamlessly between straight and turning motions. Voß and Wittkowski^78^ explored how geometric factors, particularly aspect ratios, influence acoustic propulsion for cone-shaped microswimmers. Dillinger et al.^79^ fabricated a starfish-inspired artificial cilia microswimmer, which was made of magnetized soft polymer material. This swimmer can achieve efficient acoustic propulsion due to the cilia structures, while allowing magnetic field-guided directional movement. Zhang et al.^80^ combined acoustic standing waves and rotational magnetic fields to guide magnetic microswarms along virtual acoustic walls. Their innovative approach allowed precise bidirectional rolling in an open acoustic chamber without physical boundaries, resulting in 2D dynamic manipulation with switching the orientation of virtual walls. Accordingly, their finding highlights the critical role of symmetry breaking caused by the time-varying acoustic radiation force for inducing rolling motion.

An exciting advance for acoustic microrobots is the successful application of these microrobots to *in vivo* systems. Ahmed and co-workers demonstrated several strategies of achieving acoustic-controlled motion in animal’s vasculature. Fonseca et al.^81^ reported that lipid microbubbles can self-assemble and propel under acoustic stimulation. Utilizing a combination of 18 transducers, the lipid microbubble assemblies were able to navigate through complex artificial vasculature networks. Finally, they demonstrated that these assemblies can also move against bloodstreaming in vessels of a mouse brain (Figure 3B). Deng et al.^82^ fabricated a microrobot with double helix geometries, which enabled propulsion with the self-rotation of the microrobot. By adjusting the activation frequency, the direction of the robot can be switched to enable more flexible and straightforward control of its motion. This robot enables complex motion in artificial blood vessels without the need of multiple piezoelectric transducers.

## Advances in Fluid Manipulation

Acoustofluidics is also a versatile tool for manipulating fluid at microscale. It has been used in fluid mixing and pumping for various applications, which are mainly achieved through the control of acoustic streaming, a steady fluid flow induced by the dissipation of acoustic energy in the fluid. Acoustic streaming in typical microfluidic devices is mainly boundary-driven streaming, which can be solved using Nyborg’s perturbation method. However, when the second-order acoustic streaming velocity is fast, numerical results based on the perturbation method could deviate from experimental observation significantly. Therefore, there have been significant efforts in modeling fast acoustic streaming in recent years. Friends and co-workers^83^ developed a new method to supplant the classic approach. The fast and slow spatiotemporal scales are defined based on the intrinsic properties of the fluid under forcing but not arbitrary assumption in their model. Dubrovski et al.^84^ then developed a method for arbitrary Reynolds number flow. Recently, Joergensen et al.^85^ developed models to explore the nonlinear thermoviscous effects, revealing that frictional heat could alter the streaming pattern qualitatively at high acoustic energy density.^86^ Streaming patterns are also influenced by other factors including shape of confined liquid, IDT configuration, design of microfluidic channel, operating frequency, incident position and angle, and particles in the fluid.^87^ Quelennec et al.^88^ studied the slip dynamics at the interface of liquid and solid for acoustofluidic devices. Their finding indicated that slip dynamics depends on the shear stress and fluid pressure of the system and could change as the fluid properties change.

### Sharp-Edge Fluid Manipulation

The interaction between high-speed oscillating structures with sharp edges and liquid can also induce strong acoustic streaming that is often several orders of magnitude faster than typical Rayleigh boundary-driven streaming. Due to its high efficiency and flexibility, it has gained significant popularity in recent years since the early demonstrations of mixing and pumping by Huang and co-workers.^89^ Typical sharp-edge based devices are comprised of a microchannel with sharp-edge structures that extrude out from the channel wall into the channel. Upon activation by acoustic waves, two fast rotating vortices appear on both sides of the sharp-edge structures. Pavlic et al.^90^ reported a hybrid model to improve the efficiency of sharp-edge device modeling. Due to the large discrepancy in scale between sharp-edge structures and the rest of the device, it is often difficult to balance model accuracy and efficiency for sharp-edge devices. They employed the Fully Viscous modeling approach to model the fluid domain near sharp-edge structures, while using an efficient modeling method in the rest of a device, reducing the computation demand without sacrificing the accuracy of sharp-edge simulation. Pourabed et al.^91^ reported efficient mixing using a lotus-shaped sharp-edge structure. This device achieved mixing efficiency from 80 to 94% up to 1400 μL/min flow rate in 2 ms within a mixing length of 70 μm. Pavlic et al.^92^ reported a chemically robust silicon–glass microfluidic chip with sharp-edge structures. They achieved programmable fluid pumping, mixing, cell focusing, and trapping in one device. Agha^93^ explored the integration of acoustic micromixing with cyclin olefin copolymer microfluidics. Cyclic olefin copolymers and other similar polymers are used due to their unique properties, low cost, and versatile fabrication methods. The sharp-edge mixing platform was entirely made of cyclic olefin copolymer which was fabricated in a solvent swelling process. Lu et al.^94^ combined trapped oscillating bubbles and sharp-edge structures to further enhance the streaming in microfluidic channel for efficient fluid mixing and cell lysis. In addition to standard sharp-edge structures, Harley et al.^95^ examined the impact of 3D sharp-edged microstructure geometry on acoustic streaming. Their results indicated that arbitrarily designed 3D microstructures can influence the direction and behavior of 3D streaming vortices as well as the orientation of oscillating sharp edges, allowing for both in-plane and out-of-plane vortex orientations. Furthermore, their approach showed potential to improve particle manipulation by amplifying and arbitrarily orienting fluid vortices and shear forces. Lu et al.^96^ demonstrated an acousto-inertial microfluidic chip integrating sharp edges and contraction–expansion array structures to create strong microvortices for efficient mixing and particle capturing. Operating over a wide flow range and at low actuation voltages, the platform achieved effective particle trapping, with particles exhibiting stable recirculating motion under combined acoustic and inertial forces.

In addition to using microfabricated sharp-edge structures, Li et al.^97^ introduced a vibrating sharp-tip capillary that can induce strong acoustic streaming without the need for microfabrication. Due to the highly localized activation and the glass material, the vibrating sharp-tip capillary showed excellent energy efficiency, achieving efficient fluid mixing with a power consumption as low as 1.4 mW. In addition, fluid pumping and droplet generation have also been demonstrated with a single vibrating sharp-tip capillary. Li et al.^98^ studied the influence of liquid inside the vibrating glass capillary on the streaming generated, showing that the liquid presence changed the streaming patterns and resulted in larger streaming velocity. Durrer et al.^99^ reported on a robot-assisted acoustofluidic end effector (RAEE) by connecting a piezoelectric-driven capillary to a robotic arm, enabling efficient generation of acoustic streaming around the capillary tip. Thus, this flexible device achieved droplet merging, bidirectional liquid pumping, and automated mixing of viscous liquids within a 96-well plate. They further utilized the acoustic streaming generated by a vibrating sharp-tip capillary to achieve zebrafish rotation in an imaging chamber.

### Pumping

**Figure 4:**
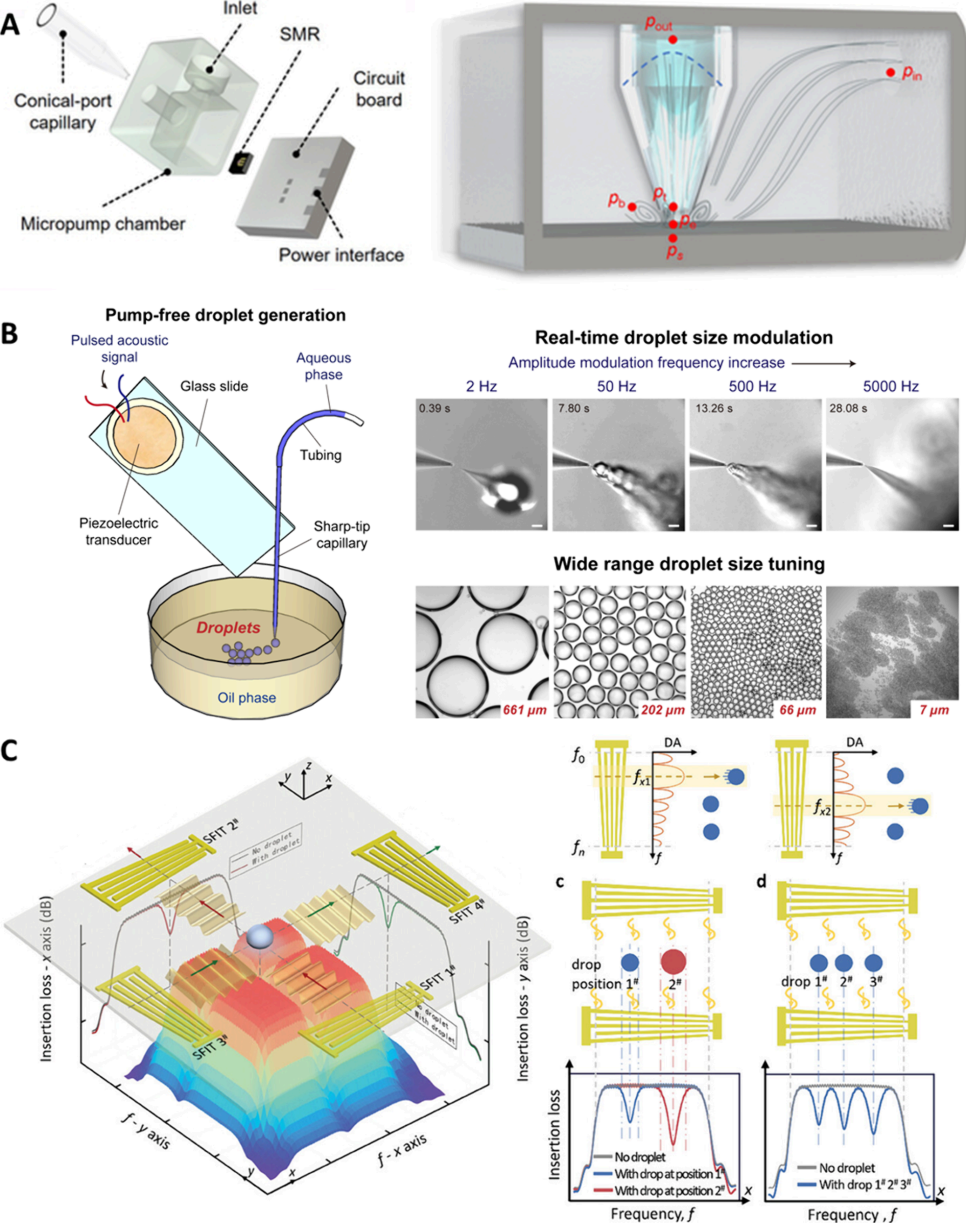
(A) A wireless pumping device based on a GHz resonator induced acoustic streaming. Reprinted by permission from Science and Technology Review Publishing House, You, R.; Fan, Q.; Wang, Z.; Xing, W.; Wang, Y.; Song, Y.; Duan, X.; Wang, Y. A Miniaturized Wireless Micropump Enabled by Confined Acoustic Streaming. *Research* 2024, 7, 0314. (ref (100)) Copyright 2024 under a Creative Commons Attribution 4.0 International License (https://creativecommons.org/licenses/by/4.0/). (B) A vibrating sharp-tip capillary for on-demand generation of water-in-oil droplets. Reprinted from *Biosens. Bioelectron.*, Vol. 191, He, Z.; Wang, J.; Fike, B. J.; Li, X.; Li, C.; Mendis, B. L.; Li, P., A Portable Droplet Generation System for Ultra-Wide Dynamic Range Digital PCR Based on a Vibrating Sharp-Tip Capillary. (ref (109)). Copyright 2021, with permission from Elsevier. (C) A closed loop droplet manipulation platform based on SIDTs. Reproduced from Acoustofluidic Tweezers Integrated with Droplet Sensing Enable Multifunctional Closed-Loop Droplet Manipulation. Sui, M.; Dong, H.; Mu, G.; Yang, Z.; Ai, Y.; Zhao, J. *Advanced Science* 2024, 2409394, (ref (118)) Copyright 2024 under a Creative Commons Attribution 4.0 International License (http://creativecommons.org/licenses/by/4.0/).

You et al.^100^ reported a wireless acoustic pumping device based on a film BAW resonator. The resonator was cut into a piece of 1*1 mm^2^ and made in direct contact with liquid to ensure high energy efficiency for pumping (Figure 4A). Upon activation by GHz RF signals, a strong Eckart jet can be induced for achieving unidirectional fluid flow. Due to its small size and wireless operation mode, it has been used for precise delivery of solution for ocular disease treatment in a pilot human trial. Mendis et al.^101^ reported a negative pressure pump for microfluidic devices based on atomization induced by a vibrating sharp tip. This setup achieved a flow rate range of 3–520 μL/min, which is dependent upon the inner diameter of the capillary tip. This setup also allows operating multiple independent pump units to achieve complex fluid operations including conducting a complete ELISA assay. Yetiskin et al.^102^ reported 3D printed acoustofluidic pumping comprised of a transducer, fluid reservoir, and a metal sheet with conical holes serving as nozzles. Oscillations were amplitude-dependent, and the motion of the metal sheet allowed the fluid to move from the reservoir to the outlet, with flow rates surpassing 515 μL/min.

## Advances in Droplet Manipulation

Various droplets have seen increased utility for diverse applications during the past decade. Aerosols are important targets for drug delivery, mass spectrometry, and environmental chemistry. Small emulsion droplets (<1 mm) are excellent microreactors that enable single biomolecule reactions and accelerate numerous organic reactions. For sub-cm droplets, they are the convenient holder for many biological samples, enabling fast and direct analysis of their content. Over the years, acoustics has been demonstrated as a powerful technique for manipulating droplets at all scales.

### Atomization

SAW can be used to atomize liquids into fine droplets. Cortez-Jugo et al.^103^ demonstrated a SAW-based platform for nebulizing small interfering RNA (siRNA) therapeutics. This platform uses high-frequency (∼10 MHz) and low-power (∼2 W) acoustic waves to generate aerosol droplets with a mean size of 2.7 μm, suitable for deep lung deposition. Huang et al.^104^ introduced a SAW device with SU-8 microchannels to nebulize salbutamol solution into monodisperse aerosol droplets for effective lung deposition to treat asthma. By optimizing input power, frequency, and flow rates, the device achieved continuous atomization with 75% lung deposition efficiency and minimized thermal degradation. Yang et al.^105^ introduced an edge-effect SAW atomizer that uses a hydrophilic paper strip as a passive capillary transport system, eliminating the need for complex fluid delivery mechanisms. The system used lateral acoustic wetting to transfer liquid from the wetted paper strip (beneath the substrate) to the piezoelectric substrate edge, where Rayleigh SAWs (40 MHz) atomized it. Roudini et al.^106^ developed a compact aerosol generator based on SAW. Using dry film photoresist (DFR) technology for microfluidics integration, the method facilitated wafer-scale fabrication of SAW chips, improving durability, scalability, and mechanical stability.

For high-viscosity fluids, Sharma et al.^107^ developed a microelectromechanical system (MEMS)-based vibrating mesh atomizer (VMA) integrated with a microheater. The localized heating reduced liquid viscosity, enabling efficient atomization with a power consumption <1 W and a temperature <100 °C. The device atomized fluids with viscosities up to 111 cP, achieving a 25-fold improvement in atomization efficiency at room temperature and a 55-fold improvement with heating.

Acoustic atomization has also been explored for propulsion systems. Amihai Horesh et al.^108^ proposed a novel approach that combines acoustothermal phase change with acoustically driven atomization to generate thrust. Their device used a 55.5 MHz focused surface acoustic wave (fSAW) to melt frozen water droplets and atomize the liquid into directed sprays to generate microscale thrust. This method achieved thrust values of up to 12.3 μN, demonstrating its potential as a propulsion solution for small satellites.

### Droplet Generation

Conventional acoustic-based droplet generation methods mainly focused on generating liquid droplets at the interface of liquid and gas. He et al.^109^ utilized the strong acoustic streaming vortices and the pumping effect generated by a vibrating sharp-tip capillary to achieve on-demand generation of monodisperse water-in-oil droplets (Figure 4B). This device dramatically reduced the equipment needed to generate size-tunable droplets, enabling portable droplet generation. They demonstrated high dynamic range digital PCR and digital LAMP leveraging the size tunability of the device. In a similar setup, Yin et al.^110^ reported that droplet ejecting direction can be controlled by adjusting the input acoustic frequency, achieving simultaneous droplet generation and dispensing. Zhou et al.^111^ reported a nozzle-free device for generating water-in-oil droplets. They employed a GHz BAW transducer to induce high pressure at the interface of water and oil, which led to the deformation of the interface with subsequent droplet pinch-off. The droplet size of the device can also be tuned by adjusting the duration and input power of the GHz acoustic signal. Steinacher and Amstad^112^ utilized SAW-induced atomization to generate water-in-oil droplets. Aqueous droplets containing Tween 80 were atomized by SAW and sprayed into an oil bath (dodecane), where emulsification was driven by a positive thermodynamic spreading coefficient. The technique enabled tunable emulsion sizes by adjusting SAW frequencies, with droplet sizes reduced from 5.3 μm at 32.5 MHz to ∼2.7 μm at 65 MHz. The process achieved stable water-in-oil emulsions at a rate of up to 480 μL/min.

### Droplet in a Closed Channel

In addition to droplet generation, many acoustofluidic systems have been reported to manipulate microdroplets inside microfluidic channels. De Lora et al.^113^ reported a device that combined acoustophoretic and dielectrophoretic forces. This system utilized embedded serpentine interdigitated transducers positioned directly next to a T-junction microchannel. Droplets flow through the main channel while a second solution is positioned perpendicular to the channel. When the acoustic and electric forces were applied, the second solution was injected into the droplets and the solutions were mixed in the droplets. Shen et al.^114^ accelerated the mixing inside microdroplets by activating a GHz transducer as the droplet passes through the channel. Kim et al.^115^ reported a method of adjusting the concentration of reagents before droplet generation. Their system, consisting of a reusable transducer and disposable microchip, used slanted finger interdigital transducers (SFITs) to form SAWs. The applied acoustics caused the two reaction solutions to mix in varying amounts depending on the flow rates of the two solutions as well as the acoustic signal. Malik et al.^116^ demonstrated that acoustic waves can also be used to modify droplets that had been generated. They designed a microfluidic chamber that can achieve droplet trapping, coalesce, and further split up, which resulted in the modification of droplet size after generation. Shakya et al.^117^ studied the SAW-based manipulation of disk-in-sphere endoskeletal droplets. They found that the orientation of the disk inside the droplet could also be tuned by changing the frequency of SAW.

### Droplet on Surface

Acoustofluidic methods have also been demonstrated to manipulate subcm droplets on a surface as well as the contents inside the droplets. One common setup for droplet manipulation is achieved by placing a droplet in the propagation path of SAW. Sui et al.^118^ developed an integrated droplet sensing and manipulation platform using slanted finger interdigital transducers (SFIDTs) (Figure 4C). The SFIDTs served as both droplet actuation unit and the droplet sensing unit. Thus, closed-loop droplet manipulation including droplet merging, transportation, and particle enrichment was achieved. Qian et al.^119^ developed a SAW platform with slanted IDTs to achieve flexible droplet manipulation. Their device consists of four slanted IDTs arranged in a square around the reaction area. They demonstrated the fusion of two droplets and subsequent centrifugation in the fused droplet. Vernon et al.^120^ reported an integrated SAW system for droplet manipulation controlled by a Raspberry Pi system. This system allows for control of droplet position, mixing, dilution, and temperature control, achieving PCR with small volumes (∼10 μL).

Liu et al.^121^ reported an acoustic black hole device to manipulate microparticles inside a droplet. The acoustic black hole was fabricated on a PMMA substrate, which can trap acoustic energy propagating on the surface of the substrate. When a droplet was placed in the acoustic black hole region, efficient particle translation and enrichment were achieved inside the droplet. Vachon et al.^122^ fabricated silicon-based membrane waveguides on a SAW droplet manipulation device. Using the waveguide, multiple droplet operations such as transportation, aggregate formation, and rotation by controlling the on/off and the frequency of RF signals applied to different IDTs. It has been reported that SAW can induce a strong centrifugal effect in a droplet for concentrating nanoparticles. Gu et al.^123^ introduced an acoustofluidic centrifuge leveraging SAW-induced droplet spinning to create rotational vortex fields, achieving rapid nanoparticle enrichment with over 80% purity and separating exosome subpopulations within a minute, offering a compact and resource-efficient alternative to ultracentrifugation. Huang et al.^124^ developed a numerical model that also considers the deformation of the droplet during spinning. Based on the simulation results, they found the particles are concentrated at a certain height above the surface, which was then confirmed with their experimental observation.

For large droplets, surface properties have a major impact on their manipulation. Several studies exploit the surface property for droplet manipulation. Yuan et al.^125^ utilized phase array transducers to trap and transport droplets on a superhydrophobic surface. Similarly, Luo et al.^126^ utilized a single ultrasonic transducer for droplet transportation followed by Raman spectroscopy detection on a superhydrophobic surface. Wu et al.^127^ utilized a SAW-induced capillary wave in a thin polymer film to fabricate a surface with different functionalities including droplet transportation, water collection, and droplet mixing.

## Advances in Applications

### Cell Separation/Enrichment

Magnusson et al.^128^ conducted a pilot study comparing the isolation performance of circulating tumor cells (CTCs) from prostate cancer patients between a BAW cell separation device with the FDA-approved method CELLSEARCH. Their results showed that the BAW device was able to isolate CTC clusters from patient samples, while no CTC clusters were detected with CELLSEARCH. This result indicated the great potential of acoustofluidic separation methods for further expanding the coverage of different types of CTCs from patient samples.

**Figure 5:**
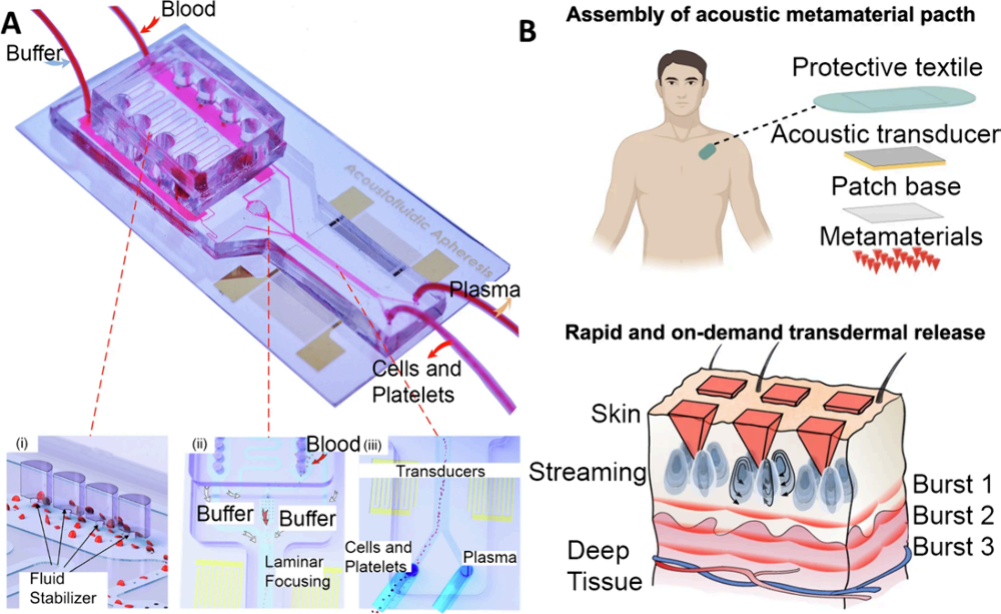
(A) An acoustic apheresis system for small blood volumes. Reprinted by permission from Macmillan Publishers Ltd.: NATURE, Wu, M.; Ma, Z.; Xu, X.; Lu, B.; Gu, Y.; Yoon, J.; Xia, J.; Ma, Z.; Upreti, N.; Anwar, I. J.; Knechtle, S. J.; T. Chambers, E.; Kwun, J.; Lee, L. P.; Huang, T. J. Acoustofluidic-Based Therapeutic Apheresis System. *Nat. Commun*. **2024**, 15 (1), 6854. (ref (129)). Copyright 2024 under a Creative Commons Attribution 4.0 International License (http://creativecommons.org/licenses/by/4.0/). (B) Pyramid structures with a piezoelectric transducer to control transdermal drug delivery. Reprinted by permission from Macmillan Publishers Ltd.: NATURE, Xu, J.; Cai, H.; Wu, Z.; Li, X.; Tian, C.; Ao, Z.; Niu, V. C.; Xiao, X.; Jiang, L.; Khodoun, M.; Rothenberg, M.; Mackie, K.; Chen, J.; Lee, L. P.; Guo, F. Acoustic Metamaterials-Driven Transdermal Drug Delivery for Rapid and on-Demand Management of Acute Disease. *Nat. Commun*. **2023**, 14 (1), 869. (ref (145)). Copyright 2024 under a Creative Commons Attribution 4.0 International License (http://creativecommons.org/licenses/by/4.0/).

Acoustofluidic devices have also been used for diverse blood sample processing applications. Wu et al.^129^ reported a therapeutic apheresis system for processing small blood volume, which is urgently needed for infants or small animals (Figure 5A). They utilized taSSAW to separate blood cells and platelets from plasma to remove pathogenic substances and antibodies. This system demonstrated successful plasmapheresis in mouse models with a requirement of only 280 μL of blood. Ma et al.^130^ leveraged impedance mismatch in an acoustofluidic device for separating platelet-reduced plasma, achieving 85–95% platelet removal with a throughput of 20 μL/min, demonstrating superior performance compared to traditional methods. Alsved et al.^131^ developed a label-free, flow-through method for isolating peripheral blood mononuclear cells (PBMCs) from undiluted blood using ultrasonic standing waves. The barrier medium and premix methods enhanced PBMC enrichment by factors of 3,600 to 11,000, with 85% separation efficiency, processing up to 20 μL/min of blood. Liu et al.^132^ utilized different sized aptamer-modified polystyrene microspheres to separate dengue virus (DENV) and tick-borne encephalitis virus (TBEV) using TSAW. Hao et al.^133^ reported a two-stage separation method for isolating nanometer-sized biomarkers of Alzheimer’s disease using SAW. First, the large micrometer-sized debris is separated from the other smaller components. Next, the nanometer-sized biomarkers of interest are separated from any remaining larger particles. Haque et al.^134^ proposed a finger-actuated device with an acoustofluidic micromixer and a manual pump, which enabled efficient RBC lysis from finger-prick blood samples (∼20 μL) in 30 s. This device, tested on ten donor samples, outperformed traditional manual assays, showing potential for scalable point-of-care diagnostics.

### Extracellular Vesicle (EV)

Rufo et al.^135^ introduced the flocculation via orbital acoustic trapping (FLOAT) method for high yield and rapid isolation of EVs. EVs are promising for a noninvasive diagnosis of disease but are extremely difficult to separate from biological samples. This method used an acoustofluidic droplet centrifuge to rotate and heat droplets. A thermoresponsive polymer was then added, allowing for nanoparticles to be rapidly and selectively concentrated at the droplet center. The use of FLOAT reduces the biofluid starting volume by a factor of 100 demonstrating its potential for point-of-care diagnostics. Naquin et al.^136^ presented acoustic separation and concentration of exosomes for nucleotide detection (ASCENDx), which allows acoustic concentration of exosomes from plasma samples. Integrated plasmonic nanostars on the disc enable label-free detection via Raman scattering. Results showed that colorectal cancer biomarkers were detected with high sensitivity and specificity. Wang et al.^137^ applied acoustofluidic exosome separation for early diagnosis of traumatic brain injury (TBI). The device can isolate exosomes which carry biomarkers indicative of TBI from blood samples. The isolation step reduced the interference for the detection of exosome biomarkers of TBI using flow cytometry. Dumcuis et al.^138^ introduced a dual-wave acoustofluidic centrifuge for the ultrafast concentration of nanoparticles and extracellular vesicles. It is difficult to determine the concentration of EVs because of the complex matrices. This method used a thin-film printed circuit board with interdigitated electrodes mounted on piezoelectric substrates. The dual wave creates an acoustic field resulting in the concentration of the cells which occurs roughly every 30 s. Wang et al.^139^ used acoustofluidics to simultaneously load nanoparticles with drugs and encapsulate exosomes. Encapsulating exosomes and nanocarriers is essential to increasing the efficacy of targeted drug delivery. To accomplish this, the device leverages acoustic radiation force, acoustic microstreaming, and shear stress in a rotating droplet. This system allows for drug loading and encapsulation to be done in minutes without the need for chemical modification.

### Intracellular Delivery

In recent years, acoustofluidics has been reported as an effective tool to facilitate the delivery of exogenous agents into cells. The thermal and mechanical effects induced by acoustic waves were found to induce transient opening of cell membrane facilitating intracellular delivery. Liu et al.^140^ reported an efficient transfection device based on the acoustothermal effect. Both SAW-induced acoustic force and heating contributed to the enhanced cell membrane permeability for gene transfection. They demonstrated the transfection of two genes simultaneously to mesenchymal stem cells (MSCs) with an efficiency of ∼90%. Salari et al.^141^ reported an acoustofluidic device for delivering cargos with a size range of 3–500 kDa. The strategy is based on the combination of acoustic streaming and lamb waves induced acoustic excitation to facilitate the uptake of different size cargos by adherent cells culture on the channel bottom surface. Gao et al.^142^ employed a phase shifting keying (PSK) technique to dynamically change the SSAW field pattern in a fluid chamber. As a result, cells trapped by the SSAW field were subject to contact oscillatory motion. The combined effect of ARF and acoustic streaming facilitated the update of drug molecules by cells. Centner et al.^143^ compared the performance of acoustofluidic systems with a static system for molecular delivery to T cells. Their system uses acoustic waves to create pressure gradients to direct molecules while a static system uses ultrasound waves without any active fluid flow. The acoustofluidic system showed similar delivery efficiency while having lower microbubble concentration and shorter treatment time. Aghaamoo et al.^144^ reported an acoustic shear orbiting platform (AESOP) to optimize intracellular delivery. This platform combines acoustic waves and electric fields to generate microvortices that facilitate the efficient entry of large molecules into cells. The micro vortices cause fluid shear stress to break down the barriers, while the electric fields create an electroporation effect to help the cell membrane become permeable. Results showed successful intracellular delivery of a range of molecule sizes (<1 kDa to 2 MDa) with high efficiency and cell viability.

Beyond the cellular level, acoustofluidics has also been used for facilitating transdermal drug delivery. Xu et al.^145^ fabricated active acoustic metamaterials with pyramid structures attached to a piezoelectric transducer (Figure 5B). This device was able to achieve precise control of the dosage and release kinetics of therapeutic reagents and demonstrated effective treatment for allergy-induced anaphylaxis in mouse models. Zhang et al.^146^ studied flexible SAW-facilitated transdermal drug delivery. Their results showed that the flexible SAW device enabled transdermal delivery of fluorescent molecules up to 2000 kDa. Wang et al.^147^ studied the blood brain barrier as it is the gatekeeper to the central nervous system, which was accomplished using an open-source board-based acoustofluidic transwell system. The acoustic waves in this system temporarily disrupted the blood brain barrier, opening it up for the desired substances to cross.

### Cell Pairing/Fusion

Zhong et al.^148^ reported a multiparametric cellular immunity analysis by modular acoustofluidic platform, CIAMAP, to manipulate immune cells using acoustic forces. This platform sorts and collects effector targets and cell pairs and monitors real-time dynamics while also having a modular design allowing for easy adaptation to different needs. In addition to understanding disease, acoustofluidics has been used to potentially help improve the treatment of them. Liu et al.^149^ used oscillating bubbles to enable rapid cell pairing and fusion. The monodisperse microbubbles were generated using multiple rectangular structures at the sidewall of the PDMS. Within 40 s, the cells were captured and paired on the surface of these bubbles. Results showed that cell membrane fusion was achieved within 20 min and that biological functions of the cells were not altered.

### Organoids Fabrication and 3D Cell Culture

Acoustofluidics is also a powerful technology for manipulating and assembling biological and synthetic systems. Ao et al.^150^ utilized SSAW to fabricate tumor spheroids using patient-derived cells. Their device can yield hundreds of cell assemblies in a Petri dish under 2 min, which can then be used for evaluating the response of different therapeutic agents. In the following work, they utilized this method to evaluate cancer immunotherapy for primary tumor-derived organotypic cell clusters (POCCs). This method maintains immune and stromal components of the TME which enables rapid evaluation of T-cell-mediated cytotoxicity and immune checkpoint inhibitor (ICI) therapies within 12 h. Shan et al.^151^ fabricated spiral transducers to generate an acoustic vortex for rapid formation of 3D tumor organoids. They also found that the acoustic field enhanced ion channel activation for Ca^2+^, accelerating intercellular adhesion for cell assembly formation. In the coculture experiment for tumor organoid and T cells, a high activation rate of T cells was observed. Zheng et al.^152^ fabricated a cell spheroid array utilizing bubble-induced vortex streaming, which allows for straightforward *in situ* drug screening. Luo et al.^153^ introduced a portable and integrated acoustic device that constructs 3D cell spheroids rapidly and contact-free. The device used ultrasound to pattern cell spheroids in capillary systems. Mei et al.^154^ developed a SAW-based method to form and manipulate 3D cell clusters. In this method, SAW induced acoustic streaming in the media to generate recirculation zones that agglomerate cells into clusters. This method allows for the rapid formation of large, spherical clusters without using traditional wells, preserving intercellular communication capabilities and promoting tissue-like functionality. Acoustic levitation has also been applied to support noncontact culture environments. Rabiet et al.^155^ used acoustic levitation for the long-term culture of human 3D spheroids without physical support. In this method, ultrasonic standing waves were used to suspend and organize cells into self-assembled spheroids. In addition to cell spheroids, Gao et al.^156^ used an acoustofluidic device to assemble DNA-coated colloids (DNACCs) into hierarchical structures. By modulating acoustic wave properties such as frequency and phase, the method created controlled, multiscale assemblies of DNACCs in a robust and programmable manner. The ability to achieve external programmability prevents kinetics trapping, allowing for the formation of complex morphologies.

### Tissue Engineering

Acoustofluidic technologies have also been used in vascular and tissue engineering applications. Wu et al.^157^ used SSAWs to create vessel-on-a-chip systems. Endothelial cells were patterned in hydrogel matrices, which would be developed into vascular networks under interstitial flow stimulation. The method enables the creation of vessel networks with high-resolution geometries, high reproducibility, and vascular functionality such as vascular permeability and perfusion. Shao et al.^158^ developed an acoustofluidic-assisted direct ink writing (DIW) method to fabricate vascular scaffolds with patterned porous microstructures. Using BAWs, CaCO_3_ particles within the sodium alginate ink were aligned, extruded around a rotating rod, and cross-linked with CaCl_2_. The CaCO_3_ particles were later removed with HCl to create porous microstructures. This approach yielded 3D tubular scaffolds with a 69% improvement in pore connectivity, 8% higher in porosity, and enhanced fibroblast cell distribution with a 96% survival rate. Yin et al.^159^ employed an acoustofluidic method for creating structured cell-laden hydrogel fibers and tubules with tunable cell patterns. This method exploits acoustic resonances to prepattern cells in liquid hydrogels before UV cross-linking into stable structures that mimic natural tissue structures like muscle fibers and blood vessels. Results showed that patterned hydrogels maintained high cell viability and proliferation over 72 h. Li et al.^160^ expanded the application of acoustofluidics into the field of responsive materials by developing an acousto-photolithography technique that combines SAW patterning with photolithography, resulting in complex transformations like bending and twisting. Deshmukh et al.^161^ used a BAWs acoustofluidic device to produce hydrogel fibers with patterned cells for tissue engineering. The BAWs were used to pattern cells within hydrogels followed by photopolymerization to hold the structure in place. This device can create patterned cellular structures with precise arrangement of cells, mimicking natural tissues such as skeletal muscle. Hu et al.^162^ presented a bubble-assisted acoustic wave method for high-precision assembly of heterogeneous tissue models. This method used oscillating bubbles in an acoustic field to pattern cells, achieving spatial resolutions up to 45 μm. The method was applied to construct an *in vitro* model of hepatic lobules with endothelial and hepatic parenchymal cells, showing functional performance such as urea and albumin secretion and enzyme activity.

### Biophysics Measurement

Acoustofluidic microdevices can be used in biophysical applications such as assessing mechanical properties of cells and distinguishing phenotypes. For example, Fu et al.^163^ designed an acoustofluidic device to assess varying metastatic potential in breast cancer cells by evaluating their compressibility in a rapid and label-free manner. When the piezoelectric transducer was activated, the standing wave filed directed cells and polystyrene beads toward the central pressure node of the channel. They further applied the device to study EMT regulation in MCF-7 cells. Those with EMT upregulation showed increased cell compressibility, whereas those with EMT downregulation showed decreased cell compressibility. Wang et al.^164^ used taSSAW to phenotype different cell lines. As the input power increased, cells migrated toward the pressure node traveling as much as 271 μm across the channel region toward the node. By testing breast and lung cancer cells along with leukocytes, they could differentiate between the three types of cells as each cell type required slightly different threshold input power to achieve migration. Hu et al.^165^ fabricated a SAW device comprised of two pairs of IDTs to create pressure nodes array to multiplex the capture and trapping of HEK293 cells. They obtained dynamics of individual cells by applying power law rheology dynamics during loading and relaxation processes.

### Cell/Organism Stimulation

Yoo et al.^166^ reported the working mechanism of neuromodulation effect induced by focused ultrasound. They found that the focused ultrasound activates neuron in culture via mechanosensitive ion channel activation. When the ion channels were inhibited, reduced response of ultrasound activation was observed, indicating the key role of these channels in ultrasonic neuromodulation. Vasan et al.^167^ observed cell membrane deflection under ultrasound stimulation using high speed holographic imaging. They developed a biomechanical model predicting the change of voltage across cell membrane, which was then experimentally confirmed using a patch-clamp assay. He et al.^168^ studied the mechanobiological secretome of mesenchymal stem cells (MSCs) upon acoustic stimulation. 3D MSC aggregates were actuated by acoustic waves to enhance the secretion from MSCs. Results showed that N-cadherin was up-regulated for the MSC aggregates, which enhanced the MSC secretome. Kim et al.^169^ investigated the cytotoxic response of natural killer cells in a microreactor to SAW with the goal of enhancing the function of immune cells using a noninvasive approach. SAW-induced acoustic streaming exerted shear stress to the cells, which enhanced the immune cell function by providing a dynamic microenvironment mimicking physiological conditions. This device was able to increase cytokine secretion which is essential for immune activation and antitumor response. Mokhtare et al.^170^ reported on a SAW device to perform oocyte denudation, which is often necessary in assisted reproductive technology (ART). This device achieved >90 denudation under various operation modes. When compared with manual denudation, a similar level of survival was observed, while the acoustic device significantly reduced the labor burden of the manual procedure. Bhadra et al.^171^ utilized SAW-induced acoustic streaming to stimulate neurodegenerative disease model *Caenorhabditis elegans*. The intensity of acoustic streaming can be precisely controlled by adjusting the input power. They found less neuron loss for *C. elegans* treated with moderate intensity acoustic streaming. While the protective effect is less significant for high-intensity acoustic streaming treatment, it still showed less neuron loss compared to the control group. Kwak et al.^172^ investigated the effects of helical flow in blood vessels in a SAW dynamic flow generator. It was found that adjusting the amplitude changes the vortices and optimal parameters are needed to preserve the integrity of the endothelial cells. The results suggested that cells respond to the helical flow through mechanosensitive ion channels and that the helical flow has an atheroprotective role.

### Sperm Manipulation

Neild and co-workers^173^ reported that a 20 s ultrasound treatment enhanced the mobility of sperms from bull and human. A 15% increase in curvilinear velocity was observed for human sperm without significant change in viability and DNA fragmentation index. In a recent work, they further showed that using a high-frequency (40 MHz) ultrasound treatment led to 34% immotile sperm becoming motile.

Castro et al.^174^ used acoustic forces to manipulate sperm cells for efficient sorting and evaluating semen quality. The platform used ADMiER, an acoustically driven microfluidic rheometry platform, which is a high-throughput method so that large volumes of semen can be analyzed quickly. Gai et al.^175^ leveraged acoustic streaming flow to analyze sperm rheotaxis without a pump. They studied different device geometries to reflect varying fallopian tubes, and the results gave insight on how sperm navigate in the female reproductive tract. It was found that rheotactic sperms swim near the boundaries to overcome the flow and reach the fertilization site. Wan et al.^176^ employed oscillating bubble-induced vortex acoustic streaming to concentrate sperm for downstream analysis. Zhang et al.^177^ used SAW to concentrate sperms in a droplet to facilitate in vitro fertilization between sperm and oocyte. An acoustic device can also be used to select mobile sperms. Misko et al.^178^ reported an acoustic focusing device that showed only the high mobility sperms could “escape” the trap.

### Imaging

The advantages in contact-free manipulation and sample separation have made acoustofluidics a powerful tool in various imaging applications. As a nondestructive technique, acoustofluidics can be coupled with different optical detection techniques such as fluorescence and Raman spectroscopy. The traditional strategy is to apply acoustofluidics for the solution mixing and enrichment of target analytes, and several new applications have been reported in recent years. Park et al.^179^ applied SAW to induce the aggregation of silver nanoparticles, achieving the signal amplification for the surface-enhanced Raman spectroscopy (SERS) detection of dopamine. Kim et al.^180^ also utilized acoustofluidics to concentrate Ebola virus particles for SERS detection. In addition to biological samples, acoustofluidics is applied for the analysis of samples which need an ultraclean environment, such as extraterrestrial material. Ferretti et al.^181^ analyzed two levitated fragments of Saratov meteorite with Raman spectroscopy using an acoustic levitator for sample manipulation. The contact-free manipulation could effectively eliminate sample contamination, demonstrating the potential of acoustofluidics in contact-free and low-contamination characterization in astronomical research. Nam et al.^182^ reported acoustofluidic lysis of MDA-MB-231 cells for Raman spectrum profiling. The cell lysis was realized through mechanical collisions with polystyrene microparticles, which was actuated by an acoustofluidic device with a high frequency.

Acoustofluidics has been adopted for *in vivo* imaging and real-time monitoring. Kellerer et al.^183^ realized the real-time monitoring of osmosis in A549 lung cells and red blood cells. The cells were trapped in a spherical microchamber with an acoustofluidic device and imaged by a two-photon-excited fluorescence microscopy. Silva et al.^184^ collected the Raman spectra of live cells using acoustofluidics for cell aggregation. Ota et al.^185^ developed high-throughput 3D-imaging flow cytometry with acoustic manipulation of cells. The cell stream was focused on a semi-1D rectangular region with the same velocity for imaging by the light-sheet microscopy. Since the cells were well-confined in a narrow region, the frame rate of a high-speed sCMOS camera could be maximized, achieving a throughput of over 2000 cell/s. The authors further improved the high-throughput 3D-imaging flow cytometry technique and applied it for the comparison of the nuclear morphology of adhering and suspended cells.^186^ The results showed that the nuclei of adhering cells were smaller and less rounded.

Acoustofluidics has been used to rotate objects to achieve multiangle imaging. Liang et al.^187^ employed refractive-index (RI)-based 3D tomographic imaging techniques for the long-term spatiotemporal observation of a single cell. An acoustofluidic device was applied for the rotation of cells during culture. This study revealed the different characteristics of MCF-7 and K562 cells during normal growth, drug-induced apoptosis, and drug-induced necrosis. The sample manipulation could also be more precisely controlled by tuning the acoustofluidic parameters. Løvmo et al.^188^ developed an acoustofluidic device to trap and manipulate biological samples for high numerical aperture multi-angle imaging. By changing the strengths of the three transducers, the sample could be rotated for image acquisition from different orientations. They further combined the acoustic trap with optical coherence tomography for zebrafish and tumor spheroid imaging. The acoustic trap allows reorientation of objects thereby achieving multiangle imaging. Richard et al.^189^ combined acoustic trapping, pneumatic valves, and light sheet microscopy to achieve high-quality imaging of macrophage phagocytosis of bacteria. Acoustic trapping ensured convenient and accurate positioning of cells during multiple rounds of imaging process. Their imaging system successfully resolved phagocytosed bacteria in the macrophage. Zhang et al.^190^ leveraged SAW-induced vortex streaming to simultaneously rotate a large number of cells for multiview precytopathological screening.

Besides working as a tool for sample manipulation, the acoustofluidic device could also play a key role in the imaging system. Jin et al.^191^ developed a dual camera acoustofluidic scanning nanoscope for high-resolution imaging. Microspheres were placed on the target sample as super resolution lens to overcome the light diffraction limit and amplify the image. In this novel nanoscope, an acoustofluidic device was applied to drive multiple microspheres simultaneously for sample scanning. The images from each microsphere could be merged with a seamless image merging algorithm (alpha-blending process) to achieve a large field of view, which was usually limited in high-resolution imaging since the field of view is inversely proportional to its resolution.

### Sample Processing

Acoustofluidics has been used to achieve many sample pretreatment steps for chemical and biological analysis. Pourabed et al.^192^ reported cell lysis from whole blood samples for blood-borne pathogens. With their method, a star-shaped device was utilized for inducing acoustic streaming within a circular channel. The blood samples were then processed for a variety of tests and diseases. Husseini et al.^193^ reported a method of using SAW and sharp-edge glass microparticles to lyse cell samples in a droplet. The system consisted of a pair of focused IDTs with the SAW beams converging in the center, where a 20 μL droplet sits. When the acoustics were applied, the SAW induced strong sharp edge streaming from the glass microparticles lysed cells.

DNA fragmentation is an important step for many DNA analysis applications including sequencing. Sun et al.^194^ reported a static lysis device using sharp-edge and bubble-induced streaming, respectively. 300 bp fragments were achieved for λ-DNA samples with an actuation duration of 150 s. Li et al.^195^ combined a vibrating sharp tip with a 3D printed microdevice, achieving DNA fragmentation under a continuous flow. The vibrating sharp tip generated strong acoustic streaming that fragmented the DNA as it flowed through the channel. DNAs were fragmented into 700–3000 bp fragments with low power consumption and a flow rate up to 50 μL/min.

Chang et al.^196^ reported a method of liquefying sputum samples based on bubble-induced acoustic streaming. The liquefied samples were collected and filtered. The resulting sample was smeared on a glass microscope slide for identification of cancer cells within the sample. This process improved the detection of cancer cells and reduced the sample processing time. He et al.^197^ utilized an acoustic focusing device to enrich biological particles including cells, crystals, and bacteria from urine under a continuous flow. As the sample flew through the device, the particles became more concentrated for convenient microscopic analysis. Costa et al.^198^ introduced EchoGrid, which is a high-throughput acoustic trapping method, with the goal of enriching microplastics for analysis. It used high-frequency acoustic waves to create a grid-like structure in which the microplastics can be selectively trapped. The technique was able to double the concentration every 78 s for 23 μm particles and every 52 s for 10 μm particles. Draz et al.^199^ reported a method of staining tissue samples for cancer detection. The tissue samples were sandwiched between substrate and coverslip. Two piezoelectric transducers were applied on opposite ends on top of the device. After the dye was loaded into the staining chamber the acoustic streaming caused the dye to travel across the entire tissue sample for uniform coverage, resulting in higher signal-to-background ratio. Ang et al.^200^ reported a SAW nanosieve device to enrich bacteria from milk samples. As the sample flows through the channel, the bacteria are captured from the other components, which facilitates downstream detection of bacteria. Nilghaz et al.^201^ reported a pumpless sharp-edge device for rapid homogenization of food samples for bacteria detection. With their method, a small portion of a raspberry or strawberry was added to a buffer, and the solution was added into the microchannel. As the sample traveled along the microchannel, sharp edges induced microvortexing for mixing and breaking down of the fruit sample. The resulting DNA in the solution was then extracted and purified for real-time PCR analysis.

### Nucleic Acids Detection

**Figure 6:**
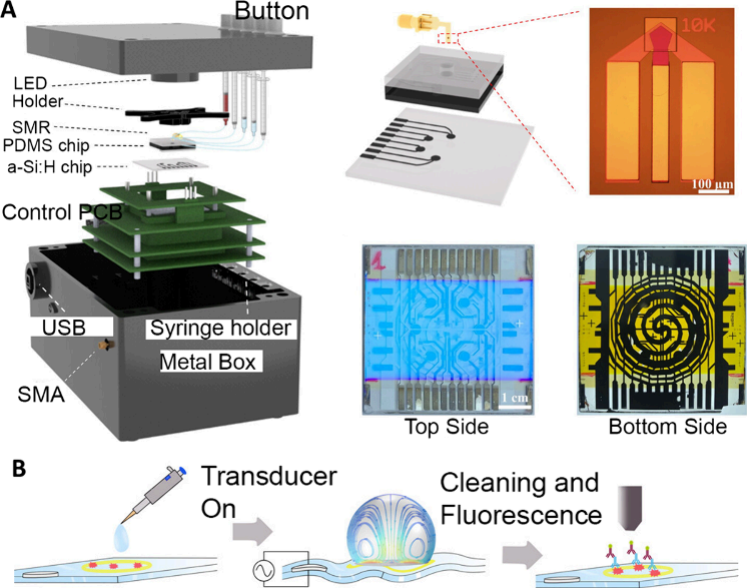
(A) An all-in-one nucleic acid detection system that has an acoustic resonator, thin-film resistors, temperature sensor, and detection module. Reproduced from Li, Z.; Li, T.; Costantini, F.; Lovecchio, N.; Chang, Y.; Caputo, D.; Duan, X. Heterogeneous Integration of Acoustic Microextraction with an Optoelectronic Sensor on Glass for Nucleic Acid Testing. *Anal. Chem*. **2024**, 96 (28), 11572–11580. (ref (204)). Copyright 2024 American Chemical Society. (B) Acoustic streaming facilitated immunoassay. Reproduced from Wang, Q.; Ding, Z.; Wong, G.; Zhou, J.; Riaud, A. Skipping the Boundary Layer: High-Speed Droplet-Based Immunoassay Using Rayleigh Acoustic Streaming. *Anal. Chem*. **2023**, 95 (15), 6253–6260. (ref (212)). Copyright 2023 American Chemical Society.

Yang et al.^202^ reported an integrated RNA detection system driven by SAW. First, cell lysis was achieved via SAW-induced collisions between cells and microparticles. Then, magnetic beads were introduced by SAW to capture the RNAs from the solution. Next, the RNAs were eluted from the magnetic beads by SAW-induced streaming. Finally, the RNA sequences were amplified in situ by PCR using SAW-induced heating. Li et al.^203^ reported an integrated microdevice for simultaneous detection of multiple respiratory pathogens. This device integrates magnetic particles-based DNA extraction and purification, acoustic streaming-enhanced fluid mixing, and PCR amplification and detection into one chip. This device leveraged oscillating bubble-induced acoustic streaming to enhance the reagents mixing after DNA extraction, achieving a limit of detection as low as 10 copies/μL. Duan and co-workers^204^ reported an all-in-one nucleic acid detection system, achieving “sample-in-answer-out”. This device integrated an acoustic resonator, thin-film resistors, temperature sensor, and detection module (Figure 6A). In this device, the acoustic unit enabled on-chip fluid manipulation and DNA extraction. Finally, this device demonstrated the detection of EGFR gene from plasma with a limit of detection (LOD) of 1 copy/μL. Zhou et al.^205^ reported that acoustic streaming inside a droplet can accelerate a recombinase polymerase amplification (RPA) reaction for detecting SARS-CoV-2. Their results showed that the acoustic streaming device halved the reaction time compared with static droplet reaction.

### Protein Detection

In addition, many studies have explored acoustic wave-induced effects to enhance protein detection. Thome et al.^206^ reported an acoustic pipet through combining functional negative acoustic contrast particles (fNACPs) and acoustic particle trapping. This acoustic pipet can directly capture and enrich biomolecules from biofluids with simple pipetting operations and enabled high-sensitivity downstream fluorescence detection. This device achieved detection of antiovalbumin antibodies from blood at pM level. Chen et al.^207^ developed a self-contained portable system for detecting total and free prostate-specific antigen using a lamb wave resonator array. The device was capable of fluid mixing, pumping, and particle trapping for analysis. The signal readout was achieved using an integrated CMOS sensor. Wu et al.^208^ employed GHz transducer-induced acoustic streaming to enrich aggregation-induced emission (AIE) probes, triggering the formation of larger aggregates. This method achieved human serum albumin detection with a LOD of 0.5 μg/mL. Das et al.^209^ utilized SAW-induced particle concentration in a droplet to accelerate the detection of protein biomarkers. It has been reported that combining acoustic trapping with a ratiometric fluorescence sensor could enhance detection signals. Zhang et al.^210^ reported a 10-fold increase in fluorescence signal when acoustic waves were used enriching the fluorophore-antibody complex. Due to the enhanced fluorescence signal and the ratiometric fluorescence sensor, semiquantitative naked eye detection was achieved. Li et al.^211^ reported using focused TSAW to trap particles for enhancing protein biomarker detection. With this method, the reaction solution flew through the reaction channel until it reached the focusing and detection area. The particles that carried target proteins got trapped in the detection area, while other particles were washed away. Wang et al.^212^ reported using Rayleigh streaming to accelerate antigen–antibody binding by eliminating the hydrodynamic boundary layer (Figure 6B). They conducted the immunoassay in a droplet placed on the SAW substrate. This method achieved much faster assay time (40 s vs 20 min) when compared to methods with the absence of acoustic streaming. Similarly, Zhang et al.^213^ reported that acoustic streaming can accelerate ELISA in a standard 96-well plate. With the assistance of acoustic streaming, each reaction step was shortened to 15 min from 60 min with conventional methods.

### Mass Spectrometry

**Figure 7:**
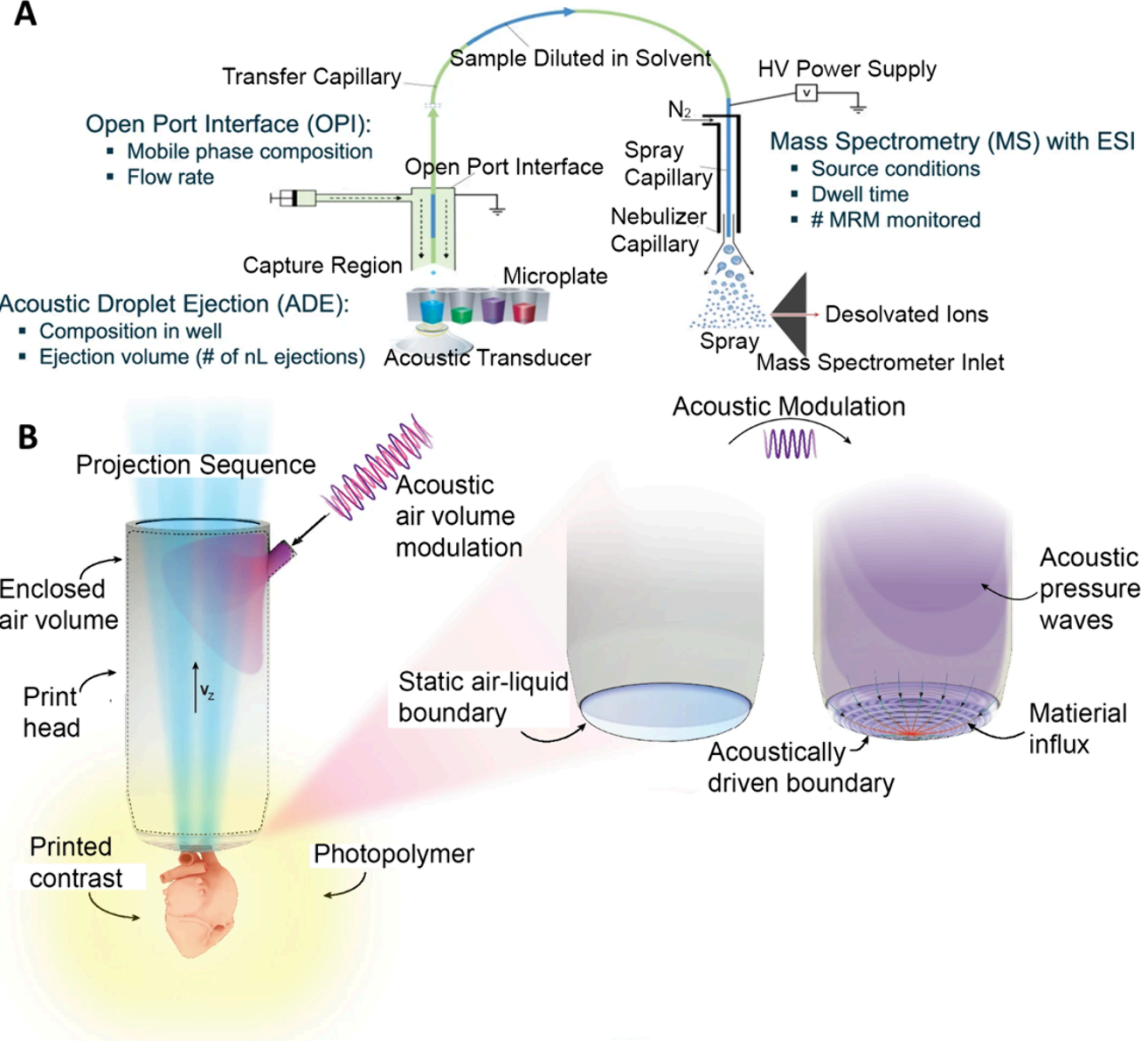
(A) Acoustic ejection-based workflow for high-throughput mass spectrometry analysis. Reprinted by permission from Macmillan Publishers Ltd.: NATURE, Van Puyvelde, B.; Hunter, C. L.; Zhgamadze, M.; Savant, S.; Wang, Y. O.; Hoedt, E.; Raedschelders, K.; Pope, M.; Huynh, C. A.; Ramanujan, V. K.; Tourtellotte, W.; Razavi, M.; Anderson, N. L.; Martens, G.; Deforce, D.; Fu, Q.; Dhaenens, M.; Van Eyk, J. E. Acoustic Ejection Mass Spectrometry Empowers Ultra-Fast Protein Biomarker Quantification. Nat. Commun. 2024, 15 (1), 5114. (ref (218)). Copyright 2024 under a Creative Commons Attribution 4.0 International License (http://creativecommons.org/licenses/by/4.0/). (B) Acoustically modulated air–liquid interfaces to facilitate 3D printing process. Reprinted by permission from Macmillan Publishers Ltd.: NATURE, Vidler, C.; Halwes, M.; Kolesnik, K.; Segeritz, P.; Mail, M.; Barlow, A. J.; Koehl, E. M.; Ramakrishnan, A.; Caballero Aguilar, L. M.; Nisbet, D. R.; Scott, D. J.; Heath, D. E.; Crozier, K. B.; Collins, D. J. Dynamic Interface Printing. *Nature***2024**, 634 (8036), 1096–1102. (ref 235). Copyright 2024.

Acoustofluidic technologies have enabled a variety of mass spectrometry applications with improved sensitivity, throughput, and efficiency. First, the acoustic ejection of droplets has significantly improved the throughput of mass spectrometry analysis. Speckmeier et al.^214^ used an acoustic droplet ejection mass spectrometry (ADE-MS) assay for screening of mutant isocitrate dehydrogenase 1 (mIDH1) inhibitors. An acoustic transducer positioned beneath a microplate rapidly ejected nanoliter droplets into a vortex carrier liquid, which transported the droplets to an electrospray ionization (ESI) source for MS analysis. This label-free approach facilitated high-throughput evaluation of enzymatic reaction directly from 384- and 1536-well plates with minimal sample preparation. It screened 3200 compounds and identified 29 potent IDH1 R132H inhibitors at 2.5 s/sample. Winter et al.^215^ employed ADE-OPI-MS for label-free, high-throughput screening of metabolic enzyme inhibitors, analyzing 54,000 compounds per day. Hu et al.^216^ also used an optimized acoustic droplet ejection-open port interface mass spectrometry method for the high-throughput analysis of oligonucleotide. The enzymatic activity of terminal deoxynucleotide transferase (TdT) was evaluated, and 10,000 TdT mutants were screened at a rate of 3 s/sample using this method. As a result, 3 new variants with 4-fold higher catalytic activity were identified. Ma et al.^217^ modified an acoustic ejection MS (AEMS) platform to enable adjustable signal durations, enabling compatibility with workflows requiring extended acquisition times. The modification allowed simultaneous monitoring of up to 13 MS/MS transitions per droplet, achieving a 10-fold throughput increase over LC-MS while maintaining high reproducibility. Puyvelde et al.^218^ also used an AEMS-based workflow for the quantification of protein biomarkers (Figure 7A). The workflow combines the speed of AEMS with the selectivity of peptide immunocapture for the high-throughput analysis of acute phase response (APR) proteins and SARS-CoV-2 peptides. As a result, the method quantified 267 plasma samples in triplicate within 4.8 h (CV: 4.2 – 10.5%) and SARS-CoV-2 peptides from 145 swabs in triplicate in just 10 min.

Cahill and Kertesz^219^ reported the rapid droplet sampling interface (RDSI) for high-throughput electrospray mass spectrometry (ESI-MS). A piezoelectric pulse was used to rapidly dispense 0.3 nL droplets into an open-face microflow capillary, where the droplets were mixed with a continuous solvent flow for electrospray ionization. The method reduced analyte dilution thereby improving sensitivity and enabled rapid sampling at 5 samples per second with fully baseline-resolved peaks.

Acoustics has also found applications in the ionization of analytes for MS analysis. Vibrating sharp-edge spray ionization (VSSI), which achieves efficiency ionization with a piezoelectric transducer and a glass slide or a capillary, has been used in many mass spectrometry applications. Wang et al.^220^ combined VSSI with a solid-phase microextraction (SPME) probe for rapid quantification of β-blockers from serum samples with a LOD of 0.25 ng/mL. The method combines desorption and ionization processes into a single step, which streamlines the analytical workflow and offers a cost-effective, robust, and portable solution for SPME-MS analysis. Pursell et al.^221^ coupled VSSI with atmospheric pressure chemical ionization (APCI) to overcome ion suppression in complex mixtures. Samples were nebulized via VSSI and then ionized via APCI. This cVSSI-APCI method improved ion signals of low-ionizing species (thymine, theophylline, and vitamin D2) in the presence of high-ionizing species (cocaine) in methanol by ∼2 to ∼3 orders of magnitude. Elshamy et al.^222^ used VSSI as a nanoflow sheath voltage-free interface to couple capillary electrophoresis (CE) to MS. Their method enabled the separation and ionization of small molecules at low flow rates under different chemical conditions. Attanayake et al.^223^ applied the field-enabled VSSI to enhance signal intensity in MS/MS experiments in negative ion mode. This technique significantly improved precursor ion intensities by more than 1000-fold and fragment ion intensities by up to 10-fold when compared with conventional ESI and HESI under the same experimental conditions. Sharif et al.^224^ reported that field-free VSSI could preserve flexible protein conformers better compared with field-enabled VSSI and ESI. In addition, VSSI has been coupled with hydrogen–deuterium exchange (HDX) and time-resolved mass spectrometry to study biomolecule structure and reaction kinetics, respectively. In addition to VSSI, Dugan et al.^225^ reported mechanospray ionization (MoSI), which achieved ionization using a piezoelectric disk and a mesh with micrometer-sized holes. Their results with biomolecules indicate that MoSI is a softer ionization method compared with ESI. Taylor et al.^226^ reported a two-stage SAW device to achieve direct analysis of lipid vesicles using mass spectrometry. The first-stage SAW used high frequency to disrupt lipid vesicles without the need for chemical reagents, which are often detrimental to mass spectrometry analysis. The second-stage SAW was a standard SAW nebulization (SAWN) device that was coupled with APCI for ionization.

Acoustics also enables a novel sample handling strategy for MS analysis. Wasen et al.^227^ used acoustic levitation combined with laser ablation and secondary electrospray ionization (SESI) for in situ MS analysis of levitated droplets. This technique uses standing acoustic waves to levitate droplets in the air to create a contactless microreactor environment. To generate the analyte plumes, levitated droplets are irradiated with a mid-IR laser, and the generated plumes are ionized with SESI for MS analysis. This method efficiently ionized and detected analytes above 1 kDa with a much-improved sensitivity compared to APCI. They further utilized acoustic levitation for real-time, contactless protein digestion analysis.^228^ The method enabled miniaturization of the digestion process, reducing the use of solvents, analytes, and enzymes. The method achieved comparable protein sequence coverage (average of 60%) to traditional overnight digestion in just 30 min. Urban and co-workers^229^ reported that placing a low-frequency (50–350 Hz) sound source close to the mass spectrometer inlet can tune the spectra obtained with ESI, which could be caused by the deflection of different sized droplets by the low-frequency sound wave.

### 3D Printing

Owing to its capability of manipulating fluids and particles without any physical contact, acoustofluidic methods have also been used to assist an additive manufacturing process. In the area of reinforced composites, Wang et al.^230^ reported acoustic-assisted 3D printing to fabricate conductive polymer composites with improved electrical properties. SSAWs created stable acoustic pressure fields that aligned silver particles and carbon fibers into various patterns such as stripes, nets, or dots within a photocurable resin matrix. These patterns were subsequently cured layer by layer using a digital light processing (DLP) projector, guided by a predefined digital mask. The resulting conductive polymer films demonstrate anisotropic electrical properties, with the highest recorded electrical conductivity of 827 S/m achieved at an optimized layer thickness of 200 μm. In advancing this concept, Xu et al.^231^ applied acoustic-assisted 3D printing to fabricate fiber-reinforced polymer composites with lightweight honeycomb structures. This method achieved a 75% increase in energy absorption and a 102% improvement in stress resistance compared to pure resin structures. Li et al.^232^ optimized the acoustic-assisted DLP method to fabricate carbon nanofiber-reinforced honeycomb structures with improved mechanical properties. By using SSAWs generated by a hexagonal array of IDTs, carbon nanofibers were aligned into strip-like patterns along honeycomb edges within a photosensitive resin. The resulting structures demonstrated significant mechanical enhancements, including an 18.1% increase in ultimate stress and a 7.9% improvement in energy absorption compared to nonreinforced resin structures. Acoustic waves have also been used to align microparticles into patterns. Xu et al.^233^ developed a multistep acoustic-assisted 3D printing method to manipulate microparticles into intricate patterns. This approach utilizes SSAW to arrange microparticles at acoustic pressure nodes within a resin matrix which is subsequently cured by a DLP projector. The method successfully fabricated two-dimensional microparticle patterns with complex geometries, such as semitriangles and hollow squares, achieving stable and precise arrangements with a minimum spacing of 200 μm. In another study, Agrawal et al.^234^ introduced a volumetric 3D printer called SonoPrint. This device is capable of patterning microparticles using acoustic waves piezoelectric transducers into lines, hexagons, and polygons within a rotating resin vial. This method enabled simultaneous reinforcement and rapid fabrication of complex structures, achieving notable mechanical improvements, including a 46% increase in tensile strength and a 13% improvement in compression strength.

Acoustic modulation has also been used to fabricate high-resolution 3D-printed structures rapidly. Vidler et al.^235^ presented a novel 3D printing technique called dynamic interface printing (DIP), leveraging acoustically modulated air–liquid interfaces to produce high-resolution structures (Figure 7B). The DIP method employs a hollow print head submerged in a photopolymer solution to create a static air–liquid meniscus, which serves as the printing interface. Capillary-gravity waves generated by acoustic modulation control material flow to this interface, where visible light polymerizes the material. This technique achieved rapid fabrication of complex geometries, including biological models such as heart, within seconds. It demonstrated print speeds of up to 700 μm/s for soft hydrogels like PEGDA, resolutions as fine as 15.1 μm, and low cytotoxicity with 93% cell viability.

### Nanoparticles Fabrication

Traditionally, ultrasound has been widely used for chemical synthesis and bulk synthesis of nanoparticles leveraging the cavitation effect. In recent years, the fast-mixing capability of acoustofluidic devices has enabled synthesizing various types of nanoparticles in microfluidic devices with high uniformity. To synthesize polymeric nanoparticles, Zhao et al.^236^ introduced a sharp-edge mixing device to fabricate PLGA–PEG nanoparticles from high molecular weight polymers (>45 kDa). This device enabled complete mixing of high molecular weight polymers like PLGA50k–PEG5k and PLGA90k–PEG10k, producing smaller PLGA–PEG nanoparticles. Additionally, this platform enabled multistep sequential nanoprecipitation, forming core–shell PLGA–PEG/lipid nanoparticles by creating a PLGA core in the first stage and assembling lipid molecules onto its surface in the second. Ozcelik et al.^237^ generates acoustic streaming flows within a low-cost glass capillary to fabricate polymer nanoparticles. Their results showed the fabrication of PLGA nanoparticles with diameters ranging from 65 to 96 nm and polydispersity index values between 0.08 and 0.18. Lu et al.^238^ combined a contraction–expansion channel with sharp-edge structures to enhance the mixing efficiency for nanoparticle synthesis. Combining the convection and acoustic mixing achieved a mixing time as low as 0.2 ms. Finally, they demonstrated the fabrication of chitosan nanoparticles with tunable sizes across a broad range of flow rates. Xu et al.^239^ utilized acoustic streaming generated around the edge of a bulk piezoelectric transducer to achieve efficient fluid mixing for fabricating liposomes. Optimized synthesis condition can be achieved by adjusting the hydrodynamic focusing of the reagent flow and the shape and orientation of a transducer. They also showed that the size of liposome can be tuned with different input power without the need to change the flow rate. Vardin et al.^240^ used a sharp-edge mixing device to synthesize liposome nanoparticles. They controlled liposome size by increasing the concentration of glycerol in the solvent, which significantly reduces the average diameter of nanoparticles. Similarly, Agha et al.^93^ synthesized liposome nanoparticles using a sharp-edge mixing platform with cyclic olefin copolymer (COC) as the channel material, a low-cost transparent biocompatible thermoplastic.

Acoustofluidics is also suitable for synthesizing metallic nanoparticles. Liu et al.^241^ utilized SAW mixing to fabricate Ag nanoparticles within a microfluidic device. Under optimal conditions and a 2:1 ratio of reductant to the precursor, the synthesized Ag nanoparticles exhibited an average size of 24.4 ± 5.1 nm. Zhang et al.^242^ used a glass capillary tube as a substrate for growing ZnO nanoarrays within the tube. They developed an acoustofluidic bubble-driven micromixer for the controllable synthesis of ZnO nanoarrays within the glass capillary. Their synthesis process involved acoustic mixing to form ZnO seeds, followed by nanorod or nanosheet growth. Another low-cost substrate for growing nanoparticles via acoustofluidics is paper. Zhao et al.^243^ demonstrates a one-step acoustofluidic method for producing plasmonic Ag nanoparticles on paper substrates. The method uses laser cutting to generate aldehydes on paper edges, serving as natural reducing agents for Ag^+^ ions, combined with acoustofluidic mixing to uniformly distribute Ag nanoparticles in 3 min. In addition to sphere nanoparticles, Curtin et al.^244^ reported a seedless and stabilizing agent-free method for synthesizing Au nanostars. They simplified the synthesis process by utilizing a 3D-printed microfluidic device with a vibrating sharp-tip acoustic mixing technique that required very low power to achieve complete mixing. Hence, high-quality Au nanostars were produced in a single-step synthesis without the need for stabilizing agents or additional postprocessing. Zheng et al.^245^ used acoustic levitated droplets for bimetallic nanoparticle synthesis. They fabricated Au–Ag nanoparticles, showing smaller size, low polydispersity, and better catalytic activity compared with traditional methods.

### Surface Cleaning

Ahmed et al.^246^ reported that SAW can effectively remove the oxidation layer on MXenes without affecting the integrity of Mxenes itself. After 1 min of SAW treatment, pseudocapacitance of the Mxenes was restored to 98% compared to 50% before treatment. Zhang et al.^247^ used a SAW-driven method for the directional clearance of bacteria captured by charged polystyrene particles. Results showed that SAW for 20 s yielded 94.8% efficiency in bacterial clearance. They also found out through motion analysis that ARF was the main factor for the clearance of bacteria. Fung et al.^248^ used oscillating sharp-edge induced acoustic streaming to clear fouling for cross-flow filtration devices. Ong et al.^249^ fabricated a ZnO thin-film SAW device on a glass substrate. Due to the transparent feature of the glass, debris removal and defogging by activating SAW is possible with such a device. In the following work, they also showed that this device can be used to inhibit the growth of bacteria.

Aerosols generated via SAW have also been leveraged for surface disinfection and decontamination. Woo et al.^250^ developed a hybrid modulation method to stabilize the nebulization of plasma-activated water (PAW) aerosols for surface disinfection and decontamination. Amplitude modulation improved nebulization rates, while pulse-width modulation alternated signal on/off cycles to reduce heating. This approach increased nebulization rates by up to 243% while preserving aerosol characteristics and reducing thermal stress. Chew et al.^251^ explored the use of SiO_2_-coated SAW devices to enhance plasma-activated aerosol generation for bacterial disinfection. The SiO_2_ coating mitigated efficiency caused by increased electrical conductivity, particularly in plasma-activated water with high reactive species concentrations. This innovation improved wettability and nebulization efficiency, achieving 76% bacterial inactivation with lower aerosol volumes and reduced treatment times.

## Conclusion and Perspectives

During the past decade, we have seen the significant growth of the acoustofluidic community, which directly led to the boom in technology development and application adoption in recent years. Numerous methods have been developed to enhance the capability of acoustofluidics in manipulating particles, fluids, and droplets across various working conditions. On the application front, we have seen the continuous expansion of acoustofluidic techniques into new areas, along with increasing demonstrations under practical and clinically relevant conditions.

Moving forward, we anticipate continued progress in several key areas:

1. Advancing Core Technologies: Continued evolution of technology to enhance the performance of existing methods and unlock new possibilities remains a top priority for the field. We believe interdisciplinary collaborations will play a crucial role in future research, enabling the integration of advanced acoustic metamaterials for fluid and particle manipulation with unprecedented resolution and precision, the development of new materials specifically optimized for acoustofluidic applications, and deeper integration with complementary technologies such as optics, DEP, magnetics, and smart materials.
2. Improving Robustness and Accessibility: Enhancing the robustness and ease of use of acoustofluidic methods is essential for broadening their impact. While the pursuit of peak performance in method development remains important, devices must also become more tolerant to real-world working conditions. Due to the nature of acoustic waves, they are inevitably affected by many factors during operation such as temperature, fluid properties, particle properties, and material properties. Multiparametric sensing and feedback systems could be considered to offer comprehensive monitoring of device operation. These systems, when combined with machine learning algorithms, can enable real-time autonomous optimization, significantly improving reliability and usability in diverse environments.
3. Discovering ″Killer Applications″: The identification and development of ″killer applications″ is critical to dramatically expanding the impact and user base of acoustofluidic methods. Such applications are the fundamental driving force for commercializing acoustofluidic technologies, which, in turn, will promote improvements in device manufacturing and operability. Building on the numerous advanced biomedical applications achieved over the past three years, we envision breakthroughs in areas such as point-of-care (POC) diagnostics, cell therapy manufacturing, and noninvasive therapeutic delivery.
